# Off-the-shelf third-party HSC-engineered iNKT cells for ameliorating GvHD while preserving GvL effect in the treatment of blood cancers

**DOI:** 10.1016/j.isci.2022.104859

**Published:** 2022-08-06

**Authors:** Yan-Ruide Li, Samuel Zeng, Zachary Spencer Dunn, Yang Zhou, Zhe Li, Jiaji Yu, Yu-Chen Wang, Josh Ku, Noah Cook, Adam Kramer, Lili Yang

**Affiliations:** 1 Department of Microbiology, Immunology & Molecular Genetics, University of California, Los Angeles, Los Angeles, CA 90095, USA; 2 Mork Family Department of Chemical Engineering and Materials Science, University of Southern California, Los Angeles, CA 90089, USA; 3 Eli and Edythe Broad Center of Regenerative Medicine and Stem Cell Research, University of California, Los Angeles, Los Angeles, CA 90095, USA; 4 Jonsson Comprehensive Cancer Center, David Geffen School of Medicine, University of California, Los Angeles, Los Angeles, CA 90095, USA; 5 Molecular Biology Institute, University of California, Los Angeles, Los Angeles, CA 90095, USA

**Keywords:** Biomedical engineering, Cell engineering, Immunology, Stem cells research

## Abstract

Allo-HSCT is a curative therapy for hematologic malignancies owing to GvL effect mediated by alloreactive T cells; however, the same T cells also mediate GvHD, a severe side effect limiting the widespread application of allo-HSCT in clinics. Invariant natural killer T (iNKT) cells can ameliorate GvHD while preserving GvL effect, but the clinical application of these cells is restricted by their scarcity. Here, we report the successful generation of third-party HSC-engineered human iNKT (^3rd^HSC-iNKT) cells using a method combining HSC gene engineering and *in vitro* HSC differentiation. The ^3rd^HSC-iNKT cells closely resembled the CD4^−^CD8^−/+^ subsets of endogenous human iNKT cells in phenotype and functionality. These cells displayed potent anti-GvHD functions by eliminating antigen-presenting myeloid cells *in vitro* and in xenograft models without negatively impacting tumor eradication by allogeneic T cells in preclinical models of lymphoma and leukemia, supporting ^3rd^HSC-iNKT cells as a promising off-the-shelf cell therapy candidate for GvHD prophylaxis.

## Highlights

• ^3rd^HSC-iNKT cells are generated off-the-shelf at high yield and purity
• ^3rd^HSC-iNKT cells ameliorate GvHD while preserving GvL effect
• ^3rd^HSC-iNKT cells effectively target antigen-presenting myeloid cells through CD1d
• ^3rd^HSC-iNKT cell product is a therapy candidate for GvHD prophylaxis in allo-HSCT

## Graphical abstract

**Figure:**
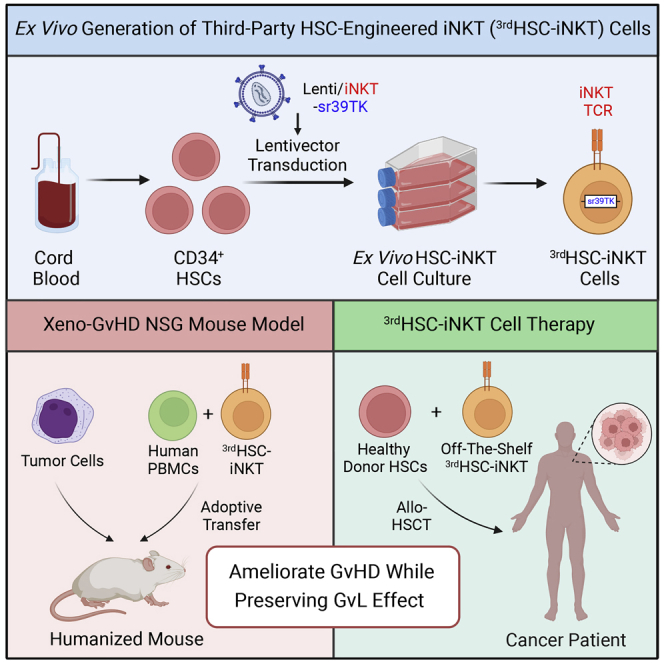

## Introduction

Allogeneic hematopoietic stem cell transplantation (allo-HSCT) is a curative therapy for hematologic malignancies such as leukemia/lymphoma owing to the graft-versus leukemia/lymphoma (GvL) effect elicited by alloreactive donor T cells (Appelbaum, 2001; Gribben and O’Brien, 2011; Shlomchik, 2007). In 2018 alone, more than 47,000 bone marrow transplantations were performed worldwide, 19,000 (41%) of which were allogeneic and nearly all for the treatment of leukemia/lymphoma (Passweg et al., 2020). However, the development of graft-versus-host disease (GvHD) mediated by alloreactive donor T cells responding to minor or major histocompatibility antigen disparities between donor and recipient remains a major cause of patient morbidity and mortality for patients receiving T-cell replete allo-HSCT (Chakraverty and Sykes, 2007; Ferrara et al., 2009; Hill et al., 2021). T cell depletion of the graft can reduce the incidence and severity of GvHD in patients but is associated with an increased risk of graft rejection, infections, and leukemia relapse (Apperley et al., 1986). Therefore, extensive research has been focused on identifying other cellular components of the graft that could modulate donor T cells and reduce the risk and severity of GvHD without diminishing normal immunological functions, including NK (Yamasaki et al., 2003), B (Shimabukuro-Vornhagen et al., 2009), and CD4^+^CD25^hi^FoxP3^+^ T regulatory (Treg) cells (Pabst et al., 2007; Wolf et al., 2007).

Invariant nature killer T (iNKT) cells have also been studied extensively for their roles in modulating GvHD and GvL. iNKT cells are a small subset of αβ T cells that express both a semi-invariant T cell receptor (TCR) (Vα24-Jα18 in humans and Vα14-Jα18 in mice paired with a limited selection of Vβ chains) and natural killer cell markers (e.g., CD161 in humans and NK1.1 in mice) (Bendelac et al., 2007; Brennan et al., 2013; Brigl and Brenner, 2004; Kronenberg, 2005; Kumar et al., 2017; Lantz and Bendelac, 1994; Taniguchi et al., 2003). Unlike conventional αβ TCRs that recognize peptide antigens presented on classical polymorphic major histocompatibility complex (MHC) Class I and II molecules, the iNKT TCR recognizes glycolipid antigens presented on non-polymorphic MHC Class I-like molecule CD1d (Cohen et al., 2009). iNKT cells in mice comprise CD4^+^ and CD4^−^CD8^−^ (double negative, DN) subsets (Brigl and Brenner, 2004), and iNKT cells in humans comprise CD4^+^, CD8^+^, and DN subsets (Brigl and Brenner, 2004). iNKT cells express high levels of cytokine mRNA and produce large amounts of cytokines on primary stimulation (Brigl and Brenner, 2004). iNKT cell subsets have differential cytokine patterns and cytolytic functions: The CD4^+^ iNKT cell subset produce much higher levels of IL-4 as compared to CD8^+^ and DN subsets; the latter subsets express much higher levels of Granzyme B and Perforin and have stronger cytolytic function as compared to the former (Brigl and Brenner, 2004).

The beneficial roles of iNKT cells in reducing GvHD while retaining GvL in murine allo-HSCT has been well reported (Lan et al., 2003; Pillai et al., 2007; Schneidawind et al., 2014; Zeng et al., 1999). Previous studies demonstrated that total lymphoid irradiation (TLI) conditioning before allo-HSCT prevented GvHD and preserved GvL effect because of the selective depletion of host conventional T cells and relative expansion of NKT cells (Lan et al., 2003; Zeng et al., 1999). The host iNKT cells interacted with donor myeloid cells to augment donor Treg expansion (Pillai et al., 2007). Addition of recipient, donor, or third-party iNKT cells into the allograft was also shown to significantly reduce the risk of GvHD in allo-HSCT without diminishing GvL by polarizing donor T cells to a Th2 phenotype (Schneidawind et al., 2014, 2015).

The protective roles of human iNKT cells against GvHD have also been highlighted by multiple clinical studies. Non-myeloablative conditioning with TLI/anti-Thymocyte Globulin (ATG) before allo-HSCT coincided with a higher iNKT/T cell ratio, decreased incidences of GvHD, and retained GvL effect (Kohrt et al., 2009; Lowsky et al., 2005). Patients with GvHD early after transplantation were found to have reduced numbers of total circulating iNKT cells (Haraguchi et al., 2004), whereas enhanced iNKT cell reconstitution following allo-HSCT positively correlated with a reduction in GvHD without loss of GvL effect (Rubio et al., 2012). In particular, high CD4^−^, but not CD4^+^, iNKT cell numbers in donor allograft was associated with clinically significant reduction in GvHD in patients receiving allo-HSCT (Chaidos et al., 2012). Thus, increasing the numbers of iNKT cells, particularly the CD4^−^ iNKT cells, in the allograft may provide an attractive strategy for suppressing GvHD while preserving GvL effect. Because of their recognition of non-polymorphic CD1d (Bae et al., 2019), iNKT cells can be sourced from third-party donors.

However, human periphery blood contains extremely low number and high variability of iNKT cells (∼0.001–1% in blood), making it challenging to expand sufficient numbers of iNKT cells for therapeutic applications (Krijgsman et al., 2018). To overcome this critical limitation, we have previously established a method to generate large amounts of human iNKT cells through TCR gene engineering of hematopoietic stem cells (HSCs) followed by *in vivo* reconstitution; using this method, we have successfully generated both mouse and human HSC-engineered iNKT (HSC-iNKT) cells (Li et al., 2021b, 2022b; Smith et al., 2015; Zhu et al., 2019). Although such an *in vivo* approach to providing iNKT cells may be suitable for autologous transplantation, applying this for allogeneic transplantation faces significant hurdles (Smith et al., 2015; Zhu et al., 2019). Here, we intended to build on the HSC-iNKT engineering approach and develop an *ex vivo* culture method to produce large amounts of third party human iNKT cells; these cells can potentially be used as a “universal” and “off-the-shelf” reagent for improving allo-HSCT outcomes by ameliorating GvHD while preserving GvL effect.

## Results

### *Ex vivo* generation and characterization of human HSC-engineered iNKT (HSC-iNKT) cells

**Figure 1:**
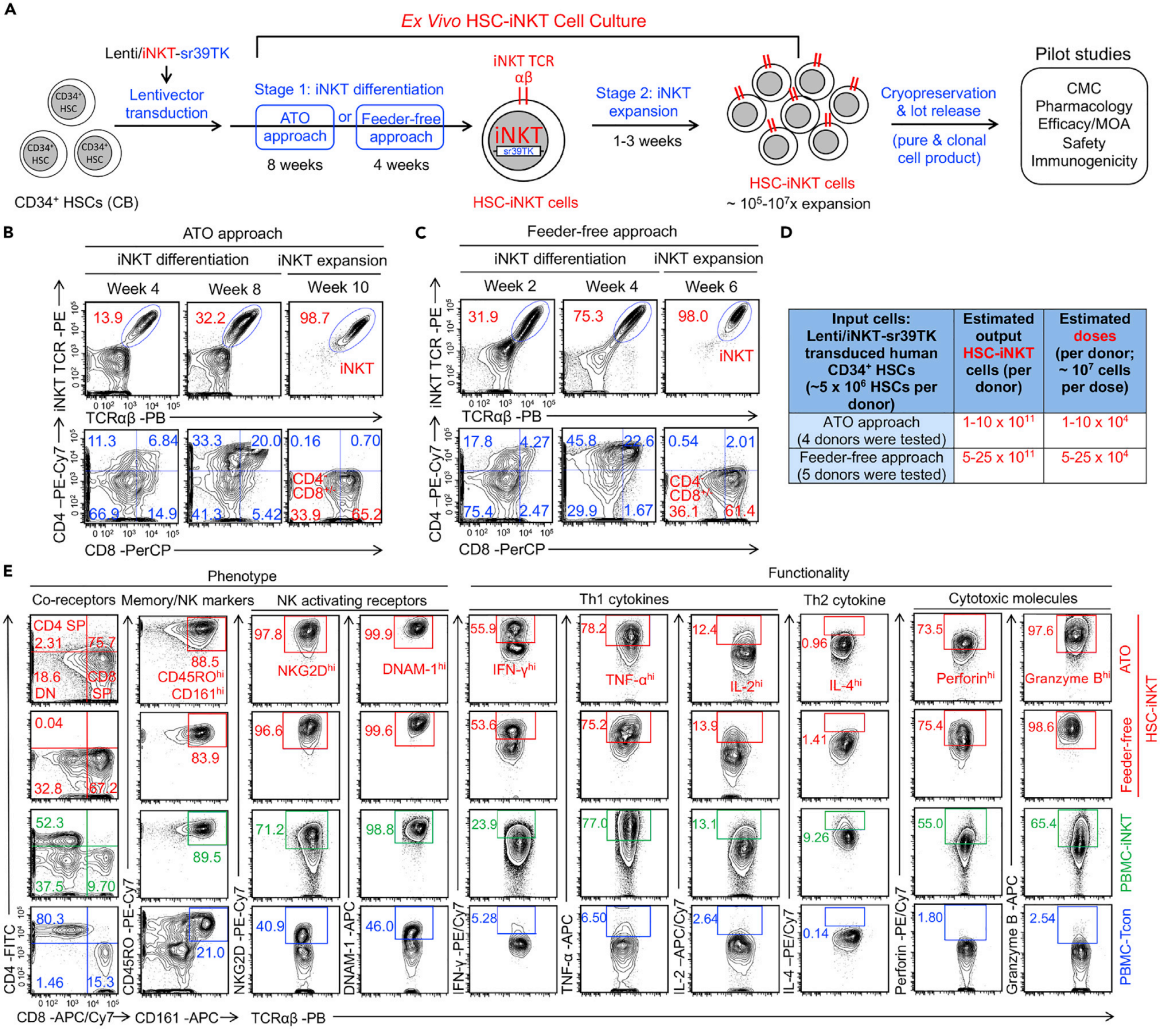
*Ex vivo* generation and characterization of HSC-engineered iNKT (HSC-iNKT) cells (A) Experimental design. HSC, hematopoietic stem cell; CB, cord blood; αGC, α-galactosylceramide; Lenti/iNKT-sr39TK, lentiviral vector encoding an iNKT TCR gene and an sr39TK suicide/PET imaging gene; ATO, artificial thymic organoid; CMC, chemistry, manufacturing, and controls; MOA, mechanism of action. (B and C) FACS monitoring of HSC-iNKT cell development during the 2-stage *Ex Vivo* HSC-iNKT Cell Culture. iNKT cells were identified as iNKT TCR^+^TCRαβ^+^ cells. iNKT TCR was stained using a 6B11 monoclonal antibody. (B) Generation of HSC-iNKT cells using an ATO approach. (C) Generation of HSC-iNKT cells using a feeder-free approach. (D) Table summarizing the production of HSC-iNKT cells. (E) FACS detection of surface markers, intracellular cytokines, and cytotoxic molecules of HSC-iNKT cells. Healthy donor periphery blood mononuclear cell (PBMC)-derived conventional αβ T (PBMC-Tcon) and iNKT (PBMC-iNKT) cells were included for comparison. Representative of over 10 experiments.

Cord blood (CB)-derived human CD34^+^ hematopoietic stem and progenitor cells (denoted as HSCs) were collected and then transduced with a Lenti/iNKT-sr39TK lentiviral vector that encodes three transgenes: A pair of iNKT TCR α and β chain genes as well as an sr39TK suicide/imaging report gene (Figure S1A) (Li et al., 2021b, 2022b; Zhu et al., 2019). The transduced HSCs were put into an *ex vivo* HSC-Derived iNKT (HSC-iNKT) cell culture, using either an artificial thymic organoid (ATO) approach or a feeder-free approach (Figure 1A). ATO culture utilizes an MS5 mouse stromal cell line overexpressing delta-like canonical Notch ligand 1 (DLL1)- or 4 (DLL4) and supports robust *ex vivo* differentiation and maturation of human T cells from HSCs (Li et al., 2021b; Montel-Hagen et al., 2019; Seet et al., 2017); feeder-free culture adopts a system of plate-bound DLL4 and vascular cell adhesion protein 1 (VCAM-1) to induce T cell commitment from HSCs (Huijskens et al., 2014; Iriguchi et al., 2021; Li et al., 2022b; Shukla et al., 2017; Themeli et al., 2013). The gene-engineered HSCs efficiently differentiated into iNKT cells in the ATO or feeder-free cultures system (Stage 1) over 8 weeks or 4 weeks, respectively, with over 100-fold expansion in cell numbers (Figures 1A–1C). These engineered HSC-iNKT cells were further expanded with irradiated PBMCs loaded with αGC, a synthetic agonist glycolipid ligand that specifically activates iNKT cells, for another 2–3 weeks (Stage 2) (Figures 1A–1C), resulting in another 100- to 1000-fold expansion of HSC-iNKT cells with >98% purity (Figures 1A and 1D). During the *ex vivo* HSC-iNKT cell cultures, HSC-iNKT cells followed a typical human iNKT cell development path defined by CD4/CD8 co-receptor expression (Godfrey and Berzins, 2007): HSC-iNKT cells transitioned from CD4^−^CD8^−^ to CD4^+^CD8^+^, then to CD4^−^CD8^+/−^ (Figures 1B and 1C). At the end of cultures, over 98% of the HSC-iNKT cells displayed a CD4^−^CD8^+/−^ phenotype (Figures 1B and 1C).

This manufacturing process of generating HSC-iNKT cells was robust and of high yield and high purity for all 9 donors tested (4 for ATO culture and 5 for feeder-free culture) (Figure 1D). Based on the results, it was estimated that from one quality CB donor (comprising about 1-5 x 10^6^ HSCs), about 10^11-^10^12^ HSC-iNKT cells could be generated that can potentially be formulated into about 10,000–100,000 doses, assuming about 10^7^ HSC-iNKT cells per dose (Figure 1D). The dosage (about 10^7^ HSC-iNKT cells per dose) was estimated based on an earlier clinical study, wherein 0.031 × 10^6^ CD4^−^ iNKT cells/kg of body weight was associated with amelioration of GvHD (Chaidos et al., 2012).

To increase the safety profile of the HSC-iNKT cell product, we included an sr39TK PET imaging/suicide gene in the lentiviral vector, which allows for the *in vivo* monitoring of these cells using PET imaging and the elimination of these cells through ganciclovir (GCV)-induced depletion in case of an adverse event (Figures S1A and S1B). In cell culture, GCV treatment induced effective killing of HSC-iNKT cells (Figures S1B and S1C). In an NSG mouse xenograft model, GCV treatment induced efficient depletion of HSC-iNKT cells from all tissues examined (i.e., blood, liver, spleen and lung) (Figures S1D–S1F). Therefore, the engineered HSC-iNKT cell product is equipped with a potent “kill switch”, significantly enhancing its safety profile.

We next studied the phenotype and functionality of HSC-iNKT cells, in comparison with healthy donor periphery blood mononuclear (PBMC)-derived iNKT (PBMC-iNKT) cells and conventional αβ T (PBMC-Tcon). HSC-iNKT cells displayed a phenotype closely resembling PBMC-iNKT cells and distinct from PBMC-Tcon cells: they expressed high levels of memory T cell markers (i.e., CD45RO) and NK cell markers (i.e., CD161, NKG2D, and DNAM-1) and expressed exceedingly high levels of Th1 cytokines (i.e., IFN-γ, TNF-α, and IL-2) as well as high levels of cytotoxic molecules (i.e., Perforin and Granzyme B) (Figure 1E). Notably, HSC-iNKT cells produced high levels of Th1 cytokines (i.e, IFN-γ, TNF-α, and IL-2) and low levels of Th2 cytokines (i.e., IL-4), suggesting a function like that of the endogenous CD8^+^ and DN human iNKT subsets, agreeing with the CD4^−^CD8^+/−^ phenotype of these HSC-iNKT cells (Figures 1B, 1C, and 1E) (Li et al., 2021b, 2022b; Zhu et al., 2019).

### Third party HSC-iNKT (^3rd^HSC-iNKT) cells ameliorate Xeno-GvHD in NSG mice engrafted with human PBMC

**Figure 2:**
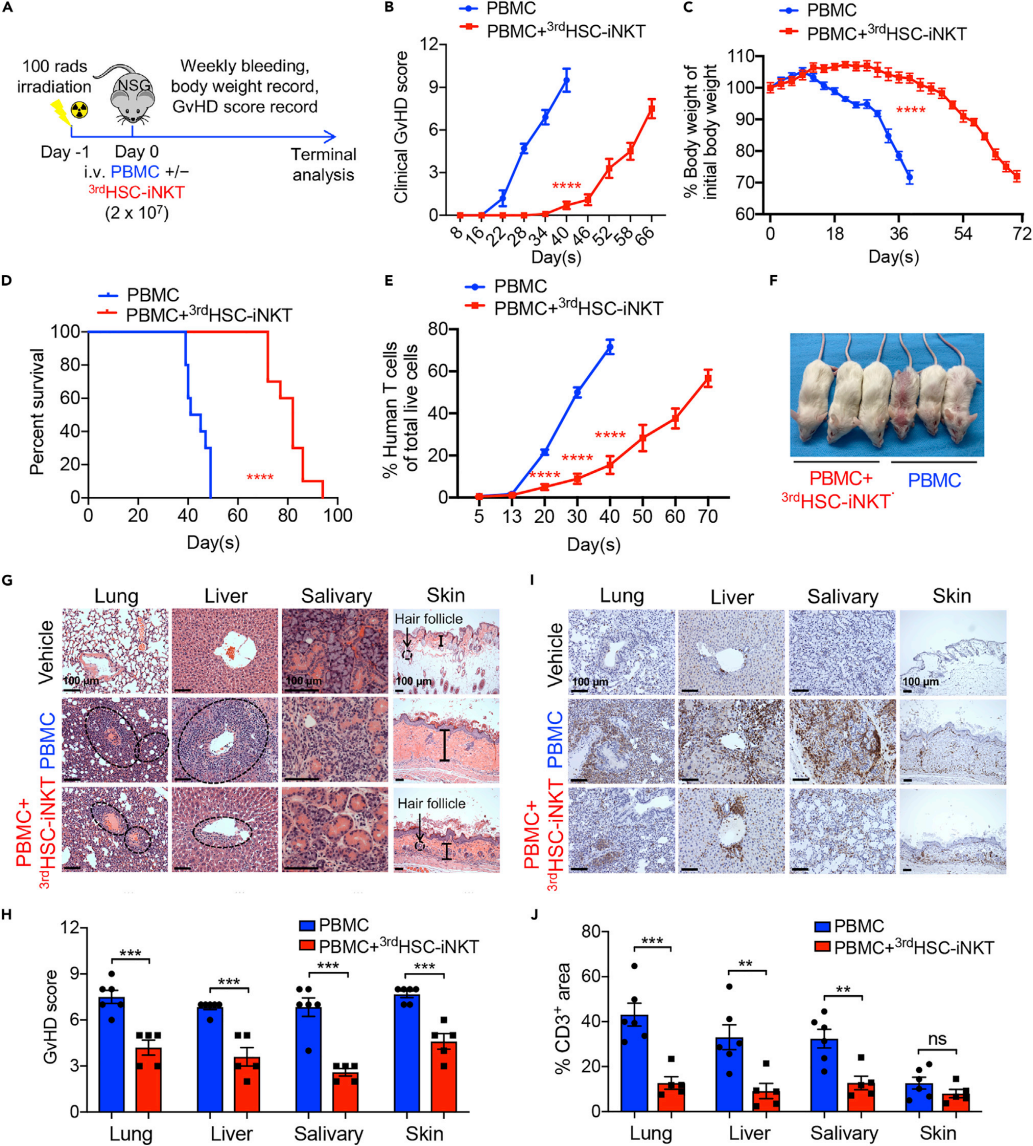
Third-party HSC-iNKT (^3rd^HSC-iNKT) cells ameliorate graft-versus-host disease (GvHD) in NSG mice engrafted with donor-mismatched human PBMCs (A–F) Sublethally irradiated NSG mice received intravenous injection of 2 × 10^7^random healthy donor PBMCs with or without the addition of 2 × 10^7 3rd^HSC-iNKT cells and were then observed for GvHD development. N = 10. (A) Experimental design. (B) Clinical GvHD score (p was calculated using data on day 40). A clinical GvHD score was calculated as the sum of individual scores of 6 categories (body weight, activity, posture, skin thickening, diarrhea, and dishevelment; score 0–2 for each category). (C) Body weight (p was calculated using data on day 40). (D) Kaplan-Meier survival curves. (E) FACS detection of human T cells in peripheral blood. (F) Representative image of experimental mice on day 40. (G–J) Histological analyses of GvHD target organs (i.e., lung, liver, salivary glands, and skin) of experimental mice analyzed 40 days following PBMC inoculation. N = 5–6. (G) H&E-stained tissue sections. Scale bar: 100 μm. (H) Quantification of (G). (I) Human CD3 antibody-stained tissue sections. Scale bar: 100 μm. (J) Quantification of (I).Representative of 3 experiments. All data are presented as the mean ± SEM. ns, not significant, ∗p< 0.05, ∗∗p< 0.01, ∗∗∗p< 0.001, and ∗∗∗∗p< 0.0001 by Student’s *t* test (B, C, E, H, and J) or by log rank (Mantel-Cox) test adjusted for multiple comparisons (D).

The engineered HSC-iNKT cells were predominantly CD4^−^ (Figures 1B, 1C, and 1E); this subset of human iNKT cells were reported to be associated with reduced GvHD in patients (Chaidos et al., 2012). To test the anti-GvHD potential of ^3rd^HSC-iNKT cells, we utilized a xeno-GvHD model wherein NSG mice were engrafted with human PBMCs (Shultz et al., 2007). NSG mice were preconditioned with non-lethal total body irradiation (TBI, 100 cGy), and were injected intravenously (i.v.) with healthy donor PBMCs with or without the addition of ^3rd^HSC-iNKT cells. The recipients were monitored daily for clinical signs of GvHD (Figure 2A). The addition of ^3rd^HSC-iNKT cells significantly delayed GvHD onset, reduced body weight loss and prolonged survival (Figures 2B–2D and 2F). The delayed onset and reduced GvHD severity were associated with the delay of donor T cell expansion in the peripheral blood of experimental mice (Figure 2E).

To further characterize the changes in GvHD severity, the acute and chronic GvHD overlapping target organs (i.e., lungs, liver, and skin) and chronic GvHD prototypical target organs (i.e., salivary glands) were collected for pathological analysis on day 40 after engrafting donor PBMCs alone or together with ^3rd^HSC-iNKT cells (Wu et al., 2013). Compared with control NSG mice, the recipient mice engrafted with PBMCs alone showed severe infiltration and damages in the liver and lung. Although the skin tissue did not have severe infiltration, there was a thickened epidermis, a sign of excessive collagen deposition (Wu et al., 2013). The salivary gland also showed infiltration and damage of gland follicles (Figures 2G–2J). These results suggested that by day 40 after PBMC engraftment, the recipient mice had overlapping acute and chronic GvHD. On the other hand, the mice receiving additional ^3rd^HSC-iNKT cells showed marked reduction in T cell infiltration in the liver, lungs, and salivary glands as well as tissue damage scores (Figures 2G–2J). Addition of ^3rd^HSC-iNKT cells also markedly reduced hair loss and epidermal thickening, although T cell infiltration in the skin tissues was mild and no significant difference was observed between recipient mice with or without the addition of ^3rd^HSC-iNKT cells (Figures 2G–2J).

Flow cytometry analysis also revealed significantly less numbers of donor T cells in the blood and spleen, as well as less T cell infiltration in GvHD target organs (i.e., lungs, liver and bone marrow; Figures S2A and S2B). Furthermore, intracellular cytokine staining showed that by day 40 after PBMC injection, the addition of ^3rd^HSC-iNKT cells significantly reduced the proportion of donor CD4^+^T cells actively producing Th1-type pro-inflammatory cytokines (i.e., IFN-γ and GM-CSF); the proportion of CD4^+^T cells producing the Th2-type anti-inflammatory cytokine (i.e., IL-4) was not changed (Figures S2C and S2D). Together, these results suggest that ^3rd^HSC-iNKT cells suppress the expansion of Th1-type pathogenic donor T cells in target tissues and thereby ameliorating acute and chronic GvHD.

### ^3rd^HSC-iNKT cells eliminate donor CD14^+^ myeloid cells in part through CD1d recognition

**Figure 3:**
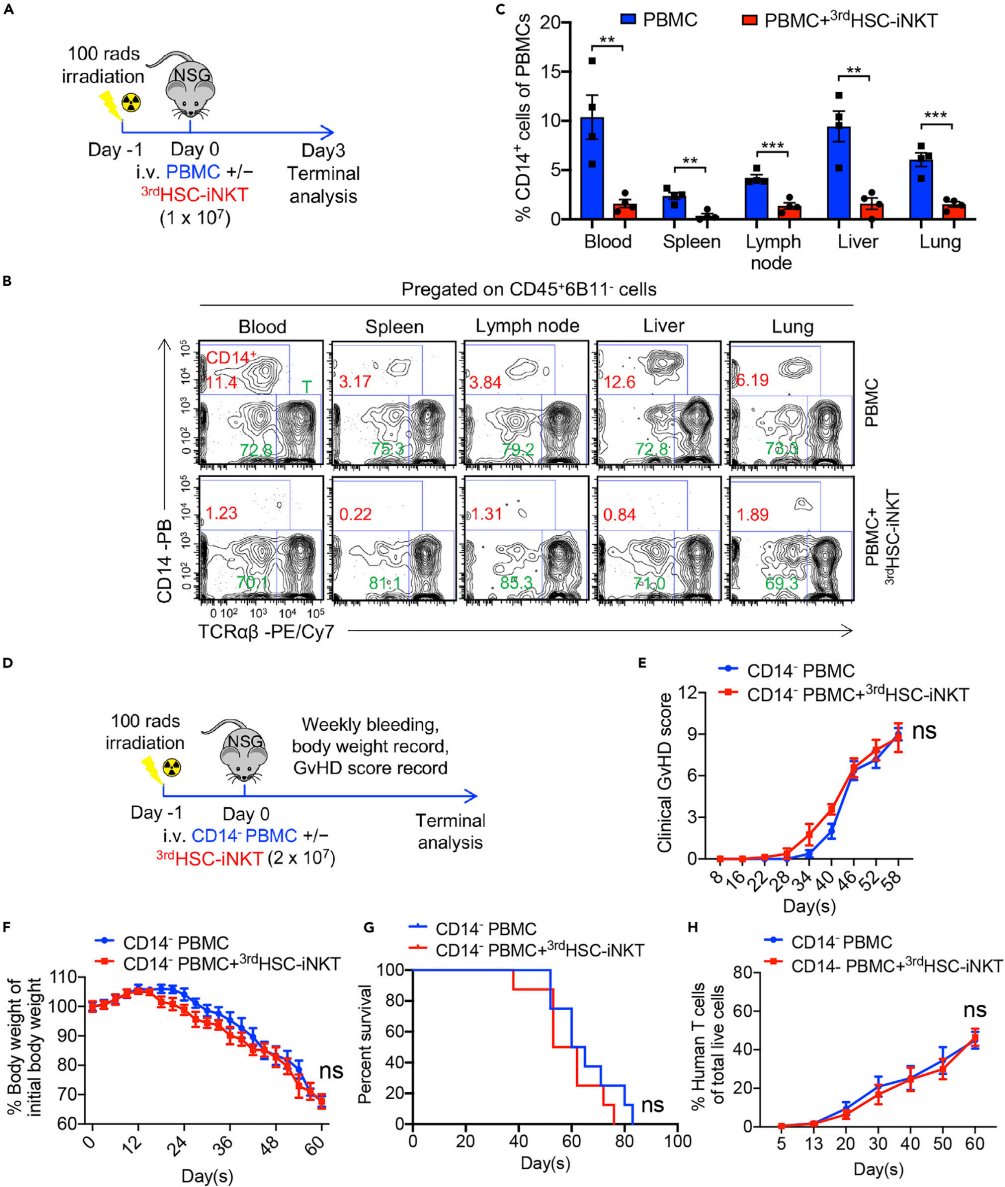
^3rd^HSC-iNKT cells ameliorate GvHD through rapid depletion of donor CD14^+^ myeloid cells that exacerbate GvHD (A–C) Sublethally irradiated NSG mice received intravenous injection of 2 × 10^7^ healthy donor PBMCs with or without the addition of 2 × 10^7 3rd^HSC-iNKT cells and were sacrificed 3 days later. (A) Experimental design. (B) FACS detection of CD14^+^ myeloid cells in the lymphohematopoietic system (i.e., blood, spleen and lymph nodes) and GvHD target organs (i.e., liver and lung). (C) Quantification of (B). N = 4. (D-H) Sublethally irradiated NSG mice received intravenous injection of 9 × 10^6^ CD14-depleted healthy donor PBMCs (matching T cell number to 2 × 10^7^ non-CD14-depleted PBMCs) with or without the addition of 2 × 10^7 3rd^HSC-iNKT cells and were then observed for GvHD development. (D) Experimental design. (E) Clinical GvHD score. (F) Body weight. (G) Kaplan-Meier survival curves. (H) Human T cells in peripheral blood. N = 8. Representative of two experiments. All data are presented as the mean ± SEM. ns, not significant, ∗p< 0.05, ∗∗p< 0.01, ∗∗∗p< 0.001, and ∗∗∗∗p< 0.0001 by Student’s *t* test (C, E, F and H) or by log rank (Mantel-Cox) test adjusted for multiple comparisons (G).

Donor myeloid cell-derived antigen presenting cells have been reported to exacerbate acute and chronic GvHD induced by donor T cells (Anderson et al., 2005; Chakraverty and Sykes, 2007; Jardine et al., 2020). Donor T cell production of GM-CSF has also been reported to recruit donor myeloid cells, which in turn amplifies the activation of allogeneic T cells and worsens GvHD severity (Piper et al., 2020; Tugues et al., 2018). Consistently, we observed that removal of CD14^+^ myeloid cells in the PBMCs reduced xeno-GvHD in NSG recipient mice (Figures 3A–3H). In contrast, co-injection of donor PBMCs together with ^3rd^HSC-iNKT cells resulted in a dramatic reduction of donor CD14^+^ myeloid cells in recipient mice within three days of injection, in tissues spanning blood, lymphoid tissues (i.e, spleen and lymph node), and GvHD target tissues (i.e., liver and lung) (Figures 3A–3C). Meanwhile, donor T and B cell, which expressed lower levels of CD1d compared to CD14^+^ myeloid cells, showed no detectable changes (Figures S4A–S4E).

iNKT cells have been shown to target myeloid (i.e., tumor-associated macrophages) and myelomonocytic cells (Cortesi et al., 2018; Gorini et al., 2017; Janakiram et al., 2017; Li et al., 2022a; Song et al., 2009). To validate that the ^3rd^HSC-iNKT cells ameliorate GvHD via the depletion of donor CD14^+^ myeloid cells in the xeno-GvHD model, we conducted another experiment wherein NSG mice received CD14^+^ myeloid cell-depleted PBMCs with or without the addition of ^3rd^HSC-iNKT cells (Figure 3D). Indeed, pre-depletion of CD14^+^ myeloid cells abrogated the anti-GvHD effect of ^3rd^HSC-iNKT cells (Figures 3E–3H).

**Figure 4:**
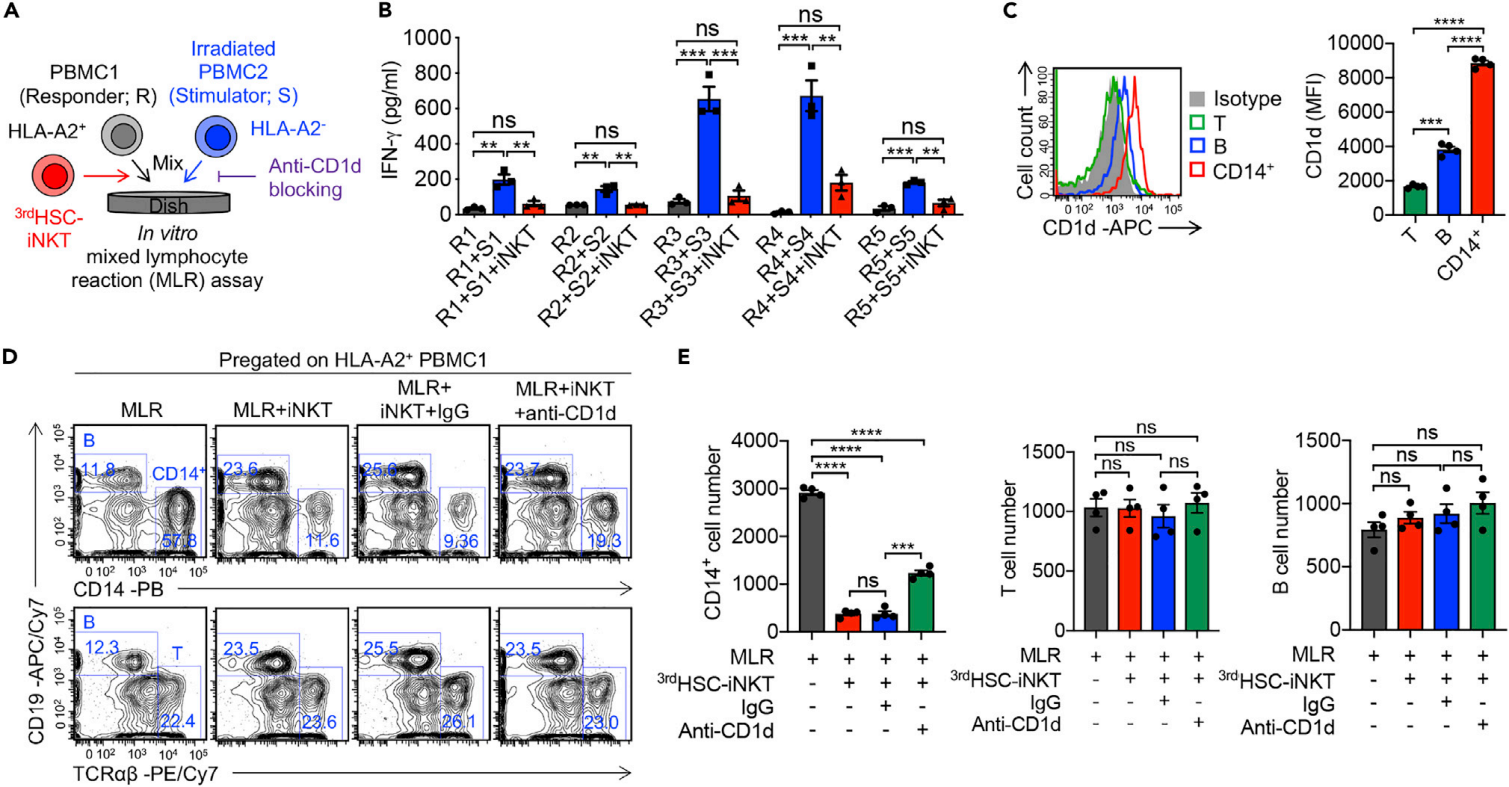
^3rd^HSC-iNKT cells ameliorate GvHD through eliminating donor CD14^+^ myeloid cells through CD1d recognition *In vitro* mixed lymphocyte reaction (MLR) assay was performed using healthy donor PBMCs (responders) co-cultured with irradiated donor-mismatched allogeneic PBMCs (stimulators) with or without the addition of ^3rd^HSC-iNKT cells. Where applicable, purified anti-human CD1d antibody or its IgG isotype control was also added. To identify responders and stimulators by flow cytometry, HLA-A2^+^ responders and HLA-A2^-^ stimulators were used in the study. (A) Experimental design. (B) ELISA analyses of IFN-γ production in the indicated MLR co-cultures. Supernatant were collected and analyzed on day 4. N = 3. (C) FACS analyses of CD1d expression on the indicated cells. N = 4. (D) FACS detection of T, B, and CD14^+^ cells of responders in multiple MLR assays one day after MLR co-culture. (E) Quantification of (D). N = 4.Representative of 3 experiments. All data are presented as the mean ± SEM. ns, not significant, ∗∗p< 0.01, ∗∗∗p< 0.001, and ∗∗∗∗p< 0.0001 by one-way ANOVA.

To study the molecular regulation of ^3rd^HSC-iNKT cell depletion of donor CD14^+^ myeloid cells, we performed an *in vitro* mixed lymphocyte reaction (MLR) assay (Figure 4A). Healthy donor PBMCs (non-irradiated; as responder representing donor cells) were mixed with donor-mismatched PBMCs (irradiated; as stimulator representing recipient cells) to study alloreaction (Li et al., 2021b), with or without the addition of ^3rd^HSC-iNKT cells. A pair of HLA-A2 positive and negative PBMCs were used to distinguish responders from stimulators (Figure 4A). In agreement with the *in vivo* results, in the MLR assay ^3rd^HSC-iNKT cells effectively ameliorated alloreaction as evidenced by the reduction of IFN-γ production (Figure 4B). Responder PBMCs contained CD14^+^ myeloid cells expressing high levels of CD1d molecule that can be recognized by iNKT TCR (Bae et al., 2019; King et al., 2018; Li et al., 2022b), corresponding to their efficient depletion by ^3rd^HSC-iNKT cells (Figures 4C–4E, S4F, and S4G). On the other hand, human T and B cells from responder PBMCs expressed low levels of CD1d and were not altered by the addition of ^3rd^HSC-iNKT cells (Figures 4C–4E). Depletion of CD14^+^ myeloid cells population was significantly alleviated by the addition of anti-CD1d blocking antibody (Figures 4D and 4E, S4F, and S4G). Taken together, ^3rd^HSC-iNKT cells ameliorate GvHD through eliminating donor CD14^+^ myeloid cells at least partly through CD1d recognition.

### ^3rd^HSC-iNKT cells preserved GvL activity while ameliorating GvHD

Next, we studied the potential of ^3rd^HSC-iNKT cells to preserve graft-versus-leukemia (GvL) while ameliorate GvHD, using a human Raji B cell lymphoma and a human HL60 acute myeloid leukemia (AML) xenograft NSG mouse models. We engineered Raji and HL60 tumor cells to overexpress the firefly luciferase and EGFP dual-reporters (denoted as Raji-FG and HL60-FG, respectively) to enable the convenient measurement of tumor killing using *in vitro* luminescence reading or *in vivo* bioluminescence imaging (BLI). When co-cultured *in vitro*, ^3rd^HSC-iNKT cells effectively killed the Raji-FG and HL60-FG cells via a NK activating receptor (i.e., NKG2D and DNAM-1)-mediated tumor targeting mechanism (Figures S5A–S5F).

**Figure 5:**
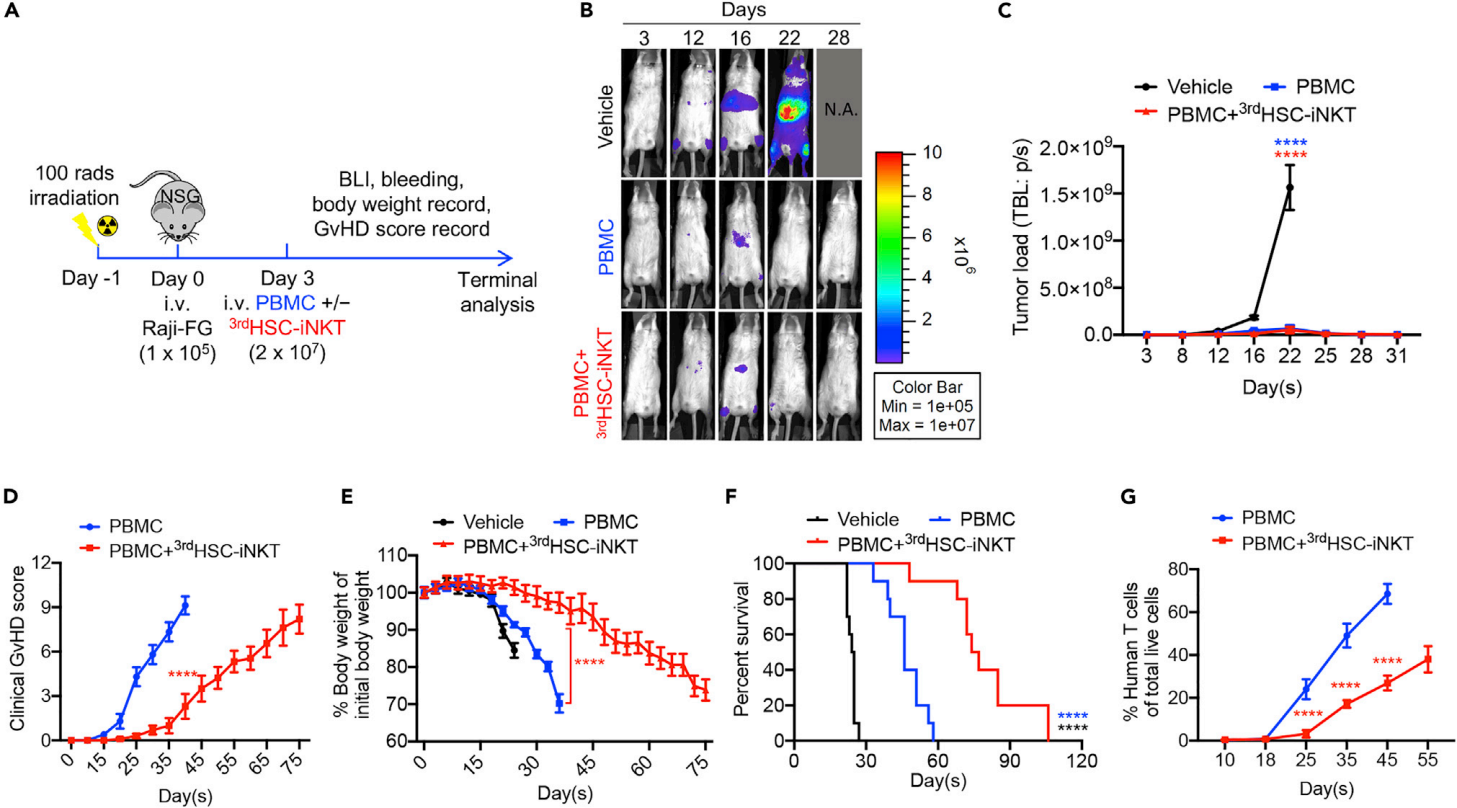
^3rd^HSC-iNKT cells ameliorate GvHD while preserving GvL in a human B cell lymphoma xenograft NSG mouse model Sublethally irradiated NSG mice were inoculated with 1 × 10^5^ Raji-FG cells, followed by intravenous injection of 2 × 10^7^healthy donor PBMCs with or without the addition of 2 × 10^7 3rd^HSC-iNKT cells. Mice were monitored for tumor burden and GvHD development. Raji-FG, human B cell lymphoma Raji cell line engineered to overexpress firefly luciferase and green fluorescence protein (FG) dual reporters. BLI, bioluminescence imaging. (A) Experimental design. (B) BLI images showing tumor loads in experimental mice over time. (C) Quantification of (B). (D) Clinical GvHD score (p was calculated using data on day 40). (E) Body weight (p was calculated using data on day 36). (F) Kaplan-Meier survival curves. (G) Human T cells in peripheral blood of experimental mice over time. N = 10. Representative of two experiments. All data are presented as the mean ± SEM. ∗∗∗∗p< 0.0001 by Student’s *t* test (D, E, G), one-way ANOVA(C), or by log rank (Mantel-Cox) test adjusted for multiple comparisons (F).

**Figure 6:**
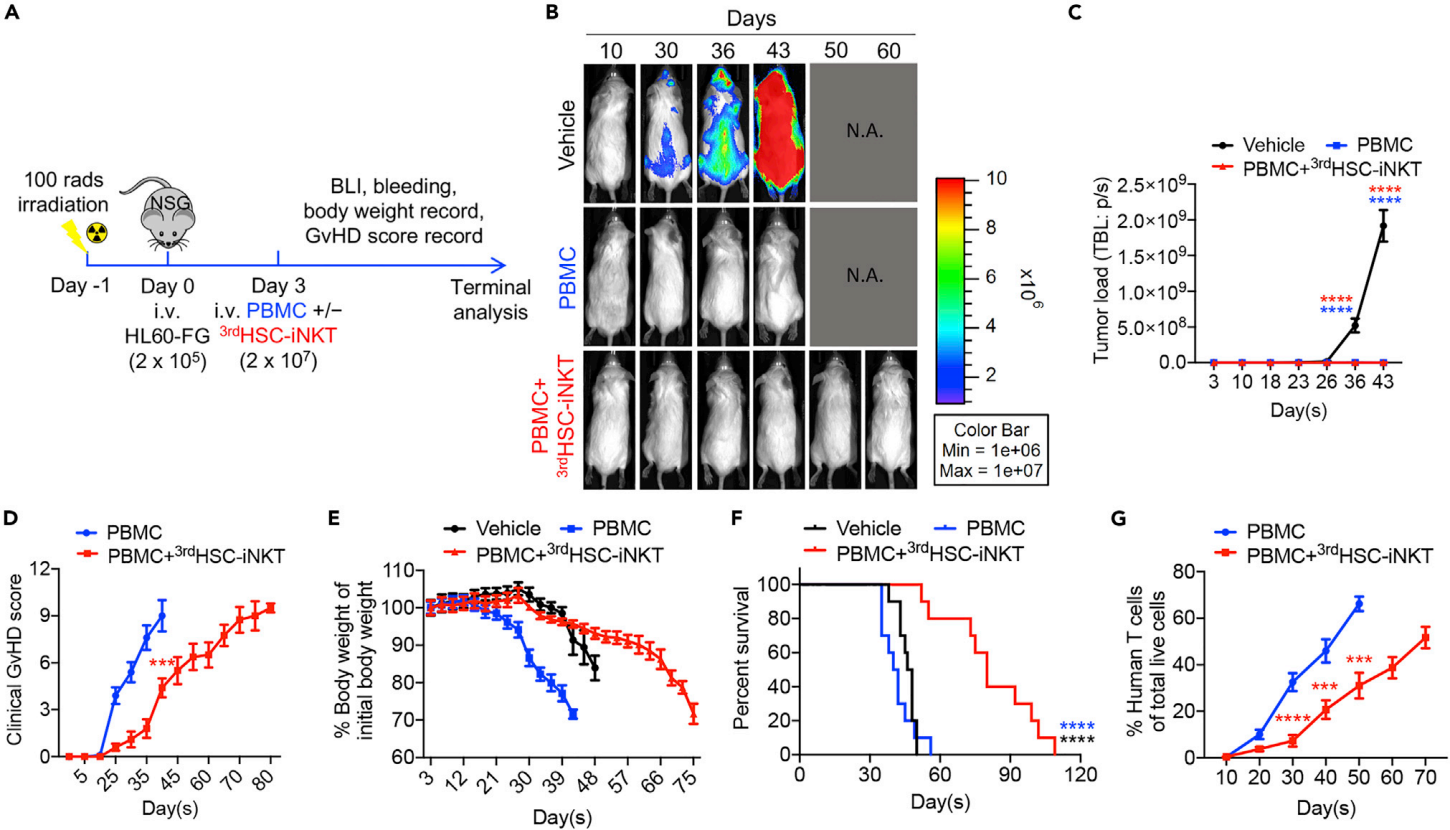
^3rd^HSC-iNKT cells ameliorate GvHD while preserving GvL in a human acute myeloid leukemia (AML) xenograft NSG mouse model Sublethally irradiated NSG mice were inoculated with 2 × 10^5^ HL60-FG human AML cells, followed by intravenous injection of 2 × 10^7^healthy donor PBMCs with or without the addition of 2 × 10^7 3rd^HSC-iNKT cells. Mice were monitored for tumor burden and GvHD development. HL60-FG, human AML HL60 cell line engineered to overexpress FG dual reporters. (A) Experimental design. (B) BLI images showing tumor loads in experimental mice over time. (C) Quantification of (B). (D) Clinical GvHD score (p was calculated using data on day 40). (E) Body weight. (F) Kaplan-Meier survival curves. (G) Human T cells in peripheral blood of experimental mice over time. N = 10. Representative of two experiments. All data are presented as the mean ± SEM. ∗∗∗p< 0.001, ∗∗∗∗p< 0.0001 by Student’s *t* test (D, G), one-way ANOVA(C), or by log rank (Mantel-Cox) test adjusted for multiple comparisons (F).

NSG mice were inoculated intravenously (i.v.) with Raji-FG cells, followed by adoptive transfer of healthy donor PBMCs without or with the addition of ^3rd^HSC-iNKT cells (Figure 5A). Control NSG mice receiving Raji-FG cells alone died as a result of high tumor burden by day 27 (Figures 5B–5F). Tumor-bearing NSG mice receiving PBMCs with or without the addition of ^3rd^HSC-iNKT cells showed rapid clearance of the Raji-FG cells (Figures 5B and 5C). However, the tumor-eradicated NSG mice receiving PBMCs all died by day 58 with high clinical GvHD scores, rapid weight loss, and rapid expansion of donor T cells (Figures 5D–5G). The mice receiving PBMCs together with ^3rd^HSC-iNKT cells survived significantly longer, for up to 106 days with a much slower progression of GvHD and decline in weight (Figures 5D–5G). Similar results were obtained from the human HL60 AML xenograft NSG mouse model (Figures 6A–6G). Taken together, these results strongly support the potential of ^3rd^HSC-iNKT cells to ameliorate GvHD while preserving GvL effect in the treatment of blood cancers.

## Discussion

iNKT cells are uniquely positioned at the crossroads of innate and adaptive immunity and have potent immunoregulatory functions in a variety of diseases (Brennan et al., 2013; Van Kaer et al., 2011). Research into harnessing iNKT cells to combat GvHD began decades ago (Lan et al., 2001), but the clinical application of iNKT cells has been hindered by their scarcity in peripheral blood (Krijgsman et al., 2018). We have recently developed an *ex vivo* HSC-iNKT culture method that can robustly generate large quantities of pure, clonal human iNKT cells (Figure 1 and S1) (Li et al., 2021b, 2022b). The resulting third-party HSC-iNKT (^3rd^HSC-iNKT) cells closely resembled peripheral blood-derived endogenous CD4^−^ iNKT cells and displayed anti-GvHD activity while preserving GvL effects in preclinical models of leukemia and lymphoma (Figures 2, 3, 4, 5, 6, and S2–S5). Importantly, such ^3rd^HSC-iNKT cells do not cause GvHD themselves and are resistant to allorejection because of their intrinsic low expression of HLA-I and II molecules (Li et al., 2021b, 2022b), highlighting their potential for off-the-shelf anti-GvHD therapy.

GvHD prophylaxis is centered around calcineurin inhibitor (CNI)-based therapy and investigations into new methods including depleting T cells, modulating T cell co-stimulatory pathways (e.g., checkpoints), enhancing regulatory T cells, targeting T cell trafficking, and altering cytokine pathways (Gooptu and Antin, 2021). Despite prophylactic interventions, acute GvHD is a common complication of allo-HSCT, occurring in 30–50% of patients, 14–36% of whom develop severe acute GvHD, and is a major cause of morbidity and mortality (Malard et al., 2020). The current first-line treatment for acute GvHD is systemic steroid therapy, but almost half of all patients will become refractory to treatment and there is no accepted standard-of-care treatment for steroid refractory-acute GvHD (Malard et al., 2020). The dismal survival rate and poor quality of life in these patients highlight the urgent need for novel therapeutic and prophylactic agents against acute GvHD.

The driver of clinical acute GvHD is donor alloreactive T cells (Ball et al., 2008). Following lymphodepletion and HSCT, host and donor antigen-presenting cells respond to host tissue damage and lead to the activation of donor T cells (Ramachandran et al., 2019). Although culpable for GvHD, HSCT-derived T cells are essential for antitumor effects, as their depletion from HSCT grafts precipitates increased relapse rates (Horowitz et al., 1990). To study the anti-GvHD potential of ^3rd^HSC-iNKT cells, we adopted an xeno-GvHD NSG mouse model, in which human PBMCs are intravenously infused and subsequent donor T cell activation results in GvHD (Figures 2 and S2), replicating some of the components of clinical GvHD (Ali et al., 2012; King et al., 2009).

Although the mechanisms are currently under investigation, our *ex vivo* culture of iNKT TCR transduced HSCs produces nearly all CD4^−^ HSC-iNKT cells (Figures 1B, 1C and 1E) (Li et al., 2021b). Like PBMC-derived endogenous CD4^−^ iNKT cells, the engineered HSC-iNKT cells express large amounts of IFN-γ and TNF-α as well as Granzyme B and Perforin (Figure 1) (Li et al., 2021b), indicative of a Th1 cytokine profile and cytotoxic potential (Li et al., 2021a, 2021b). In addition, these HSC-iNKT cells display low response to IL-12/IL-18 innate signaling *in vitro* (Data not shown). In 2012, Chaidos et al. conducted a comprehensive analysis of all immune populations in allogeneic HSCT grafts, and found that only CD4^−^ iNKT cells were correlated with reduced acute GvHD occurrence (Chaidos et al., 2012). Five years later, Rubio et al. also revealed that only pre-transplant donor CD4^−^ iNKT cells predicted clinical acute GvHD following HSCT (Rubio et al., 2017). Corroborating the clinical findings, a preclinical study from the same research team confirmed CD4^−^ iNKT cells, but not CD4^+^ iNKT cells, prevented GvHD using a xenograft NSG mouse model (Coman et al., 2018), and *in vitro* assays revealed that CD4^−^ iNKT cells reduced the maturation and induced the apoptosis of human DCs (Coman et al., 2018). In our study, ^3rd^HSC-iNKT cells ameliorated GvHD though depleting donor CD14^+^ myeloid cells, at least partly via CD1d recognition (Figures 3 and 4). Interestingly, CD4^+^ subpopulation of iNKT cells has also been implicated in GvHD amelioration, albeit through different mechanisms (Chaidos et al., 2012; Coman et al., 2018; Mavers et al., 2017; Rubio et al., 2012). The beneficial role in GvHD has been attributed to IL-4-induced Treg expansion in preclinical syngeneic mouse models (Lan et al., 2003; Pillai et al., 2007; Schneidawind et al., 2014, 2015). One interesting future direction would be modifying our *ex vivo* HSC-iNKT cell culture to produce CD4^+^ human iNKT cells to harness the anti-GvHD potential of this subpopulation of iNKT cells.

iNKT cells can also play a direct role in tumor killing. Through CD1d dependent and independent means, iNKT cells have been shown to lyse a variety of tumor cells (King et al., 2018; Li et al., 2021b; Zhu et al., 2019). Furthermore, in hematological and solid tumor models, adoptive transfer of iNKT cells reduces tumor burden and enhances overall survival (Fujii et al., 2013). Our previous studies have demonstrated the antitumor functions of HSC-iNKT cells *in vivo* when targeting CD1d positive and negative cancer cells (Li et al., 2021b; Zhou et al., 2021). Importantly, HSC-iNKT cells do not recognize mismatched MHCs and thus pose no risk of inducing GvHD; furthermore, because of their intrinsic low expression of HLA-I and II molecules, these cells are resistant to allorejection (Li et al., 2021b, 2022b). These features of HSC-iNKT cells make them suitable for allogeneic cell therapy.

Allo-HSCT is an established, effective treatment for hematological malignancies, but GvHD is a common and debilitating adverse event for many allo-HSCT recipients. We propose to develop the off-the-shelf HSC-iNKT cell therapy to ameliorate GvHD while preserving GvL in the treatment of blood cancers. The reported *ex vivo* HSC-iNKT cell culture is robust and of high yield and purity, with the potential of being scaled for further translation and clinical development. From one cord blood donor, over 10,000 doses of third-party HSC-iNKT cells can be manufactured and cryopreserved for ready distribution to allo-HSCT patients; MHC matching is not needed. This study highlights the potential of ^3rd^HSC-iNKT cells to address a critical unmet medical need and warrants further investigations of this promising off-the-shelf cell product.

### Limitations of the study

Predominant mouse models studying GvHD typically employ transplantation of T cell-depleted bone marrow and donor-derived T cells into lethally irradiated recipients; these are paramount to advance the forefront of knowledge regarding the incidence of GvHD within allo-HSCT therapeutics (Schroeder and DiPersio, 2011). In this study, healthy donor T cells were used to generate a PBMC-xenograft NSG mouse model, producing a construct where T cell-mediated GvHD could be studied and manipulated *in vivo*. However, limitations to this model preclude its ability to fully reflect GvHD pathology in allo-HSCT. Such complexity arises from factors such as the restricted availability of human embryonic tissue for transplant, the need for sublethal total body irradiation, demand for a high quantity of human PBMCs, and instability in the onset window of GvHD (Huang et al., 2018). In addition, murine immunoreaction after engraftment of human immune cells is highly distinct compared to that in humans in regard to both biological phenotype and genetics. Therefore, developing a model that more accurately mimics human GvHD pathology, while reducing variance from these limitations, is necessary to understand patient reactivity to allo-HSCT therapies.

## STAR★Methods

### Key resources table

**Table:** 

REAGENT or RESOURCE	SOURCE	IDENTIFIER
**Antibodies**		

Anti-human IFN-γ (ELISA, capture)	BD Biosciences	CAT#551221, RRID: AB_394099
Anti-human IFN-γ (ELISA, detection)	BD Biosciences	CAT#554550, RRID: AB_395472
Anti-human CD34 (Clone 581)	BD Biosciences	CAT#555822, RRID: AB_396151
Anti-human TCR Vα24-Jβ18 (Clone 6B11)	BD Biosciences	CAT#552825, RRID: AB_394478
Anti-human CD45 (Clone H130)	Biolegend	CAT#304026, RFID: AB_893337
Anti-human TCRαβ (Clone I26)	Biolegend	CAT#306716, RRID: AB_1953257
Anti-human CD4 (Clone OKT4)	Biolegend	CAT#317414, RRID: AB_571959
Anti-human CD8 (Clone SK1)	Biolegend	CAT#344714, RRID: AB_2044006
Anti-human CD45RO (Clone UCHL1)	Biolegend	CAT#304216, RRID: AB_493659
Anti-human CD161 (Clone HP-3G10)	Biolegend	CAT#339928, RRID: AB_2563967
Anti-human CD14 (Clone HCD14)	Biolegend	CAT#325608, RRID: AB_830681
Anti-human CD19 (Clone SJ25C1)	Biolegend	CAT#363005, RRID: AB_2564127
Anti-human CD11b (Clone ICRF44)	Biolegend	CAT#301330, RRID: AB_2561703
Anti-human CD1d (Clone 51.1)	Biolegend	CAT#350308, RRID: AB_10642829
Anti-human NKG2D (Clone 1D11)	Biolegend	CAT#320812, RRID: AB_2234394
Anti-human DNAM-1 (Clone 11A8)	Biolegend	CAT#338312, RRID: AB_2561952
Anti-human HLA-A2 (Clone BB7.2)	Biolegend	CAT#343308, RRID: AB_2561567
Anti-human IFN-γ (Clone B27)	Biolegend	CAT#506518, RRID: AB_2123321
Anti-human Granzyme B (Clone QA16A02)	Biolegend	CAT#372204, RRID: AB_2687028
Anti-human Perforin (Clone dG9)	Biolegend	CAT#308126, RRID: AB_2572049
Anti-human TNFα (Clone Mab11)	Biolegend	CAT#502912, RRID: AB_315264
Anti-human IL-2 (Clone MQ1-17H12)	Biolegend	CAT#500341, RRID: AB_2562854
Anti-human IL-4 (Clone MP4-25D2)	Biolegend	CAT#500824, RRID: AB_2126746
Anti-human GM-CSF (Clone BVD2-21C11)	Biolegend	CAT#502313, RRID: AB_2561838
LEAF purified anti-human NKG2D antibody (Clone 1D11)	Biolegend	CAT#320810, RRID: AB_2133276
LEAF purified anti-human DNAM-1 antibody (Clone DX11)	BD Biosciences	CAT#559786, RRID: AB_397327
Mouse IgG1, κ isotype control antibody (Clone MOPC-21)	Biolegend	CAT#400124, RRID: AB_2890215
LEAF purified anti-human CD1d antibody (Clone 51.1)	Biolegend	CAT#350304, RRID: AB_10641291
LEAF purified Mouse IgG2b, k isotype ctrl (Clone MG2b-57)	Biolegend	CAT#401201, RRID: AB_2744505
Human Fc Receptor Blocking Solution (TrueStain FcX)	Biolegend	CAT#422302, RRID: AB_2818986
Mouse Fc Block (anti-mouse CD16/32)	BD Biosciences	CAT#553142, RRID: AB_394657

**Bacterial and virus strains**		

Lenti/iNKT-sr39TK	This paper	N/A
Lenti/FG	This paper	N/A

**Biological samples**		

Human peripheral blood mononuclear cells (PBMCs)	UCLA	N/A
Cord Blood Cryo CD34	HemaCare	CAT#CB34C-3

**Chemicals, peptides, and recombinant proteins**		

Streptavidin-HRP conjugate	Invitrogen	CAT#SA10001
IFN-γ (ELISA, standard)	eBioscience	CAT#29-8319-65
Tetramethylbenzidine (TMB)	KPL	CAT#5120–0053
Ganciclovir (GCV)	Sigma	CAT#ADV465749843
Recombinant human IL-2	Peprotech	CAT#200–02
Recombinant human IL-3	Peprotech	CAT#200–03
Recombinant human IL-7	Peprotech	CAT#200–07
Recombinant human IL-15	Peprotech	CAT#200–15
Recombinant human Flt3-Ligand	Peprotech	CAT#300–19
Recombinant human SCF	Peprotech	CAT#300–07
Recombinant human TPO	Peprotech	CAT#300–18
Recombinant human GM-CSF	Peprotech	CAT#300–03
L-ascorbic acid 2-phosphate	Sigma	CAT#A8960-5G
B27™ Supplement (50X), serumfree	ThermoFisher	CAT#17504044
α-Galactosylceramide (KRN7000)	Avanti Polar Lipids	SKU#867000P-1mg
X-VIVO 15 Serum-free Hematopoietic Cell Medium	Lonza	CAT#04–418Q
RPMI1640 cell culture medium	Corning Cellgro	CAT#10-040-CV
DMEM cell culture medium	Corning Cellgro	CAT#10-013-CV
Fetal Bovine Serum (FBS)	Sigma	CAT#F2442
MACS BSA stock solution	Miltenyi	CAT#130-091-376
30% BSA	Gemini	CAT#50-753-3079
Penicillin-Streptomycine-Glutamine (P/S/G)	Gibco	CAT#10378016
Penicillin: streptomycin (pen:strep) solution (P/S)	Gemini Bio-products	CAT#400–109
MEM non-essential amino acids (NEAA)	Gibco	CAT#11140050
HEPES Buffer Solution	Gibco	CAT#15630056
Sodium Pyruvate	Gibco	CAT#11360070
Beta-Mercaptoethanol	Sigma	SKU#M6250
Normocin	InvivoGen	CAT#ant-nr-2
Cell Fixation/Permeabilization Kit	BD Biosciences	CAT#554714
RetroNectin recombination human fibronectin fragment, 2.5mg	Takara	CAT#T100B
10% neutral-buffered formalin	Richard-Allan Scientific	CAT#5705
D-Luciferin	Caliper LIfe Science	CAT#XR-1001
Isoflurane	Zoetis	CAT#50019100
Phosphate Buffered Saline (PBS) pH 7.4 (1X)	Gibco	CAT#10010–023
Formaldehyde	Sigma-Aldrich	CAT#F8775
Golgistop Protein Transport Inhibitor	BD Biosciences	CAT#554724
Phorbol-12-myristate-13-acetate (PMA)	Calbiochem	CAT#524400
Ionomycin, Calcium salt, Streptomyces conglobatus	Calbiochem	CAT#407952
Poloxamer Synperonic F108	Sigma	CAT#07579–250G-F
Prostaglandin E2	Cayman Chemical	CAT#14-190-136
Fixable Viability Dye eFluor506	affymetrix eBioscience	CAT#65-0866-14

**Critical commercial assays**		

Human CD34 MicroBeads Kit	Miltenyi Biotec	CAT#130-046-703
Human CD14 MicroBeads Kit	Miltenyi Biotec	CAT#130-050-201
Human Anti-iNKT MicroBeads	Miltenyi Biotec	CAT#130-094-842
Fixation/Permeabilization Solution Kit	BD Sciences	CAT#55474
StemSpan™ Lymphoid Differentiation Coating Material (100X)	Stem Cell Technologies	CAT#9925
StemSpan™ SFEM II	Stem Cell Technologies	CAT#9605
ImmunoCult™ Human CD3/CD28/CD2 T Cell Activator	Stem Cell Technologies	CAT#10970
Cryostor cell cryopreservation media	Sigma	CAT#C2874-100ML

**Experimental models: Cell lines**		

Human Burkitts lymphoma cell line Raji	ATCC	CCL-86
Human acute myeloid leukemia cell line HL60	ATCC	CCL-240
Human Burkitts lymphoma cell line Raji-FG	This paper	N/A
Human acute myeloid leukemia cell line HL60-FG	This paper	N/A

**Experimental models: Organisms/strains**		

NOD.Cg-Prkdcscid Il2rgtm1Wjl/SzJ (NSG)	The Jackson Laboratory	Stock #: 005557

**Recombinant DNA**		

Vector: parental lentivector pMNDW	(Giannoni et al., 2013; Lan et al., 2006)	N/A

**Software and algorithms**		

FlowJo Software	FlowJo	https://www.flowjo.com/solutions/flowjo/downloads
Living Imaging 2.50 software	Xenogen/PerkinElmer	http://www.perkinelmer.com/lab-products-and-services/resources/in-vivo-imaging-software-downloads.html
AURA imaging software	Spectral Instruments Imaging	https://spectralinvivo.com/software/
I-control 1.7 Microplate Reader Software	Tecan	https://www.selectscience.net/tecan/i-control–microplate-reader-software/81307
ImageJ	ImageJ	https://imagej.net/Downloads
Prism 6	Graphpad	https://www.graphpad.com/scientific-software/prism/

### Resource availability

#### Lead contact

Further information and requests for new reagents generated in this study may be directed to, and will be fulfilled by the lead contact, Lili Yang (liliyang@ucla.edu).

#### Materials availability

All unique/stable reagents generated in this study are available from the lead contact with a completed Materials Transfer Agreement.

### Experimental model and subject details

#### Mice

NOD.Cg-Prkdc^SCID^Il2rg^tm1Wjl^/SzJ (NOD/SCID/IL-2Rγ^−/−^, NSG) mice were maintained in the animal facilities at the University of California, Los Angeles (UCLA). Six- to ten-week-old, a mixture of male and female mice was used for all experiments. The mouse gender had no effect on our study. All animal experiments were approved by the Institutional Animal Care and Use Committee of UCLA.

#### Cell lines and viral vectors

The murine bone marrow derived stromal cell line MS5-DLL4 was obtained from Dr. Gay Crooks’ lab (UCLA). Human Raji B cell lymphoma cell line, HL60 acute myeloid leukemia cell line, and HEK 293 T cell line were purchased from the AmericanType Culture Collection (ATCC).

Lentiviral vectors used in this study were all constructed from a parental lentivector pMNDW (Li et al., 2021b, 2022b; Zhu et al., 2019). The Lenti/iNKT-sr39TK vector was constructed by inserting into pMNDW vector a synthetic tricistronic gene encoding human iNKT TCRα-F2A-TCRβ-P2A-sr39TK; the Lenti/FG vector was constructed by inserting into pMNDW a synthetic bicistronic gene encoding Fluc-P2A-EGFP. The synthetic gene fragments were obtained from GenScript and IDT. Lentiviruses were produced using HEK 293T cells, following a standard calcium precipitation protocol and an ultracentrifigation concentration protocol (Li et al., 2021b, 2022b; Zhu et al., 2019). Lentivector titers were measured by transducing HT29 cells with serial dilutions and performing digital qPCR (Li et al., 2021b, 2022b; Zhu et al., 2019).

To make stable tumor cell lines overexpressing firefly luciferase and enhanced green fluorescence protein (FG) dual-reporters, parental tumor cell lines were transduced with lentiviral vectors encoding the intended gene(s). 72h following lentiviral transduction, cells were subjected to flow cytometry sorting to isolate gene-engineered cells for making stable cell lines. Two stable tumor cell lines were generated for this study, including Raji-FG and HL60-FG.

#### Human periphery blood mononuclear cells (PBMCs)

Healthy donor human PBMCs were obtained from the UCLA/CFAR Virology Core Laboratory, with identification information removed under federal and state regulations. Cells were cryopreserved in Cryostor CS10 (BioLife Solutions) using CoolCell (BioCision) and were stored in liquid nitrogen for all experiments and long-term storage.

#### Media and reagents

α-Galactosylceramide (αGC, KRN7000) was purchased from Avanti Polar Lipids. Recombinant human IL-2, IL-3, IL-4, IL-7, IL-15, Flt3-Ligand, Stem Cell Factor (SCF), Thrombopoietin (TPO), and Granulocyte-Macrophage Colony-Stimulating Factor (GM-CSF) were purchased from Peprotech. Ganciclovir (GCV) was purchased from Sigma.

X-VIVO 15 Serum-Free Hematopoietic Cell Medium was purchased from Lonza. RPMI 1640 and DMEM cell culture medium were purchased from Corning Cellgro. Fetal bovine serum (FBS) was purchased from Sigma. Medium supplements, including penicillin-streptomycin-glutamine (P/S/G), MEM non-essential amino acids (NEAA), HEPES Buffer Solution, and sodium pyruvate, were purchased from GIBCO. beta-mercaptoethanol (β-ME) was purchased from Sigma. Normocin was purchased from InvivoGen. Complete lymphocyte culture medium (denoted as C10 medium) was made of RPMI 1640 supplemented with FBS (10% vol/vol), P/S/G (1% vol/vol), MEM NEAA (1% vol/vol), HEPES (10 mM), sodium pyruvate (1 mM), β-ME (50 mM), and Normocin (100 mg/mL). Medium for culturing human Raji and HL60 tumor cell lines (denoted as R10 medium) was made of RPMI 1640 supplemented with FBS (10% vol/vol) and P/S/G (1% vol/vol). Medium for culturing HEK 293T cell line (denoted as D10 medium) was made of DMEM supplemented with FBS (10% vol/vol) and P/S/G (1% vol/vol).

### Method details

#### Antibodies and flow cytometry

All flow cytometry stains were performed in PBS for 15 min at 4°C. The samples were stained with Fixable Viability Dye eFluor506 (e506) mixed with Mouse Fc Block (anti-mouse CD16/32) or Human Fc Receptor Blocking Solution (TrueStain FcX) before antibody staining. Antibody staining was performed at a dilution according to the manufacturer’s instructions. Fluorochrome-conjugated antibodies specific for human CD45 (Clone H130), TCRαβ (Clone I26), CD4 (Clone OKT4), CD8 (Clone SK1), CD45RO (Clone UCHL1), CD161 (Clone HP-3G10), CD69 (Clone FN50), CD56 (Clone HCD56), CD62L (Clone DREG-56), CD14 (Clone HCD14), CD1d (Clone 51.1), NKG2D (Clone 1D11), DNAM-1 (Clone 11A8), IFN-γ (Clone B27), Granzyme B (Clone QA16A02), Perforin (Clone dG9), TNF-α (Clone Mab11), IL-2 (Clone MQ1-17H12), HLA-A2 (Clone BB7.2) were purchased from BioLegend; Fluorochrome-conjugated antibodies specific for human CD34 (Clone 581) and TCR Vɑ24-Jβ18 (Clone 6B11) were purchased from BD Biosciences. Human Fc Receptor Blocking Solution (TrueStain FcX) was purchased from BioLegend, and Mouse Fc Block (anti-mouse CD16/32) was purchased from BD Biosciences. Fixable Viability Dye e506 were purchased from Affymetrix eBioscience. Intracellular cytokines were stained using a Cell Fixation/Permeabilization Kit (BD Biosciences). Stained cells were analyzed using a MACSQuant Analyzer 10 flow cytometer (Miltenyi Biotech). FlowJo software was utilized to analyze the data.

#### Enzyme-linked immunosorbent cytokine assays (ELISAs)

The ELISAs for detecting human cytokines were performed following a standard protocol from BD Biosciences. Supernatants from co-culture assays were collected and assayed to quantify IFN-γ. Capture and biotinylated pairs for detecting cytokines were purchased from BD Biosciences. The streptavidin-HRP conjugate was purchased from Invitrogen. Human cytokine standards were purchased from eBioscience. Tetramethylbenzidine (TMB) substrate was purchased from KPL. The samples were analyzed for absorbance at 450 nm using an Infinite M1000 microplate reader (Tecan).

#### *In vitr*o generation of HSC-Engineered iNKT (HSC-iNKT) cells

Cord blood-derived human CD34^+^ hematopoietic stem and progenitor cells (denoted as HSCs) were obtained from HemaCare. Frozen-thawed HSCs were revived in HSC-culture medium comprised X-VIVO 15 Serum-Free Hematopoietic Cell Medium supplemented with human recombinant SCF (50 ng/mL), FLT3-L (50 ng/mL), TPO (50 ng/mL), and IL-3 (10 ng/mL) for 24 h. Cells were then transduced with Lenti/iNKT-sr39TK viruses for another 24 h (Li et al., 2021b, 2022b; Zhu et al., 2019). The transduced HSCs were then collected and put into an Artificial Thymic Organoid (ATO) culture or a Feeder-Free culture.

In the ATO culture, transduced HSCs were mixed with MS5-DLL4 feeder cells to form ATOs and cultured over ∼8 weeks (Li et al., 2021b; Montel-Hagen et al., 2019). Briefly, MS5-DLL4 cells were harvested and resuspended in serum-free ATO culture medium (‘‘RB27”) composed of RPMI 1640 (Corning), 4% B27 supplement (Thermo Fisher Scientific), 30 mM L-ascorbic acid 2-phosphate sesquimagnesium salt hydrate (Sigma-Aldrich) reconstituted in PBS, 1% penicillin/streptomycin (Gemini BioProducts), 1% Glutamax (Thermo Fisher Scientific), 5 ng/mL rhFLT3L and 5 ng/mL rhIL-7 (Peprotech). 1.5-6 x 10^5^ MS5-DLL4 cells were mixed with 0.3–10 × 10^4^ transduced HSCs per ATO and centrifuged at 300 g for 5 min at 4°C, and the cell pellet was resuspended in 5 μL RB27 per ATO and plated on a 0.4 mm Millicell transwell insert (EMD Millipore; Cat. PICM0RG50) placed in a 6-well plate containing 1 mL RB27 per well. Medium was changed completely every 3–4 days by aspiration from around the cell insert followed by replacement with 1 mL fresh RB27/cytokines. In the feeder-free culture, transduced HSCs were cultured using a StemSpan^TM^T Cell Generation Kit (StemCell Technologies) over ∼5 weeks following the manufacturer’s instructions (Li et al., 2022b). The resulting HSC-iNKT cells isolated from ATOs or Feeder-Free culture were expanded with αGC-loaded PBMCs (αGC-PBMCs). To prepare αGC-PBMCs, 1–10 × 10^7^ PBMCs were incubated in 5 mL C10 medium containing 5 μg/mL αGC for 1 h, followed by irradiation at 6,000 rads. HSC-iNKT cells were mixed with irradiated αGC-PBMCs at ratio 1:1, followed by culturing for 2 weeks in C10 medium supplemented with human IL-7 (10 ng/mL) and IL-15 (10 ng/mL); cell cultures were split, and fresh media/cytokines were added if needed. The resulting HSC-iNKT cell products were then collected and cryopreserved for future use.

#### Generation of PBMC-Derived conventional T (PBMC-Tcon) and iNKT (PBMC-iNKT) cells

Healthy donor PBMCs were obtained from the UCLA/CFAR Virology Core Laboratory and were used to generate the PBMC-Tc and PBMC-iNKT cells.

To generate PBMC-Tcon cells, PBMCs were stimulated with CD3/CD28 T-activator beads (ThermoFisher Scientific) and cultured in C10 medium supplemented with human IL-2 (20 ng/mL) for 2–3 weeks, following the manufacturer’s instructions.

To generate PBMC-iNKT cells, PBMCs were enrich for iNKT cells using anti-iNKT microbeads (Miltenyi Biotech) and MACS-sorting, followed by stimulation with donor-matched irradiated αGC-PBMCs at the ratio of 1:1 and cultured in C10 medium supplemented with human recombinnat IL-7 (10 ng/mL) and IL-15 (10 ng/mL) for 2–3 weeks. If necessary, the resulting PBMC-iNKT cells could be further purified using Fluorescence-Activated Cell Sorting (FACS) via human iNKT TCR antibody (Clone 6B11; BD Biosciences) staining.

#### HSC-iNKT cell phenotype and functional study

HSC-iNKT cells were analyzed in comparison with PBMC-Tcon and PBMC-iNKT cells. Phenotype of these cells was studied using flow cytometry by analyzing cell surface markers including co-receptors (i.e., CD4 and CD8), NK cell receptors (i.e., CD161, NKG2D, and DNAM-1), and memory T cell markers (i.e., CD45RO). The capacity of these cells to produce cytokines (i.e., IFN-γ, TNF-α, IL-2, and IL-4) and cytotoxic molecules (i.e., Perforin and Granzyme B) were studied using flow cytometry via intracellular staining.

#### Ganciclovir (GCV) *in vitro* and *in vivo* killing assay

For GCV *in vitro* killing assays, HSC-iNKT cells were cultured in C10 medium in the presence of titrated amount of GCV (0–50 μM) for 4 days; live HSC-iNKT cells were then counted using a hematocytometer (VWR) via Trypan Blue staining (Fisher Scientific).

GCV *in vivo* killing assay was performed using an NSG xenograft mouse model. NSG mice received i.v. injection of 1 × 10^7^ HSC-iNKT cells on day 0, followed by i.p. injection of GCV for 5 consecutive days (50 mg/kg per injection per day). On day 5, mice were terminated. Multiple tissues (i.e., blood, spleen, liver, and lung) were collected and processed for flow cytometry analysis to detect tissue-infiltrating HSC-iNKT cells (identified as iNKT TCR^+^CD45^+^), following established protocols (Li et al., 2021b, 2022b; Zhu et al., 2019).

#### *In vitro* tumor cell killing assay

Tumor cells (1 × 10^4^ cells per well) were co-cultured with HSC-iNKT cells (at ratios indicated in figure legends) in Corning 96-well clear bottom black plates for 24 h, in C10 medium. At the end of culture, live tumor cells were quantified by adding D-luciferin (150 μg/mL; Caliper Life Science) to cell cultures and reading out luciferase activities using an Infinite M1000 microplate reader (Tecan).

In some experiments, 10 μg/mL of LEAF^TM^ purified anti-human NKG2D (Clone 1D11, Biolegend), anti-human DNAM-1 antibody (Clone 11A8, Biolegend), or LEAF^TM^ purified mouse lgG2bk isotype control antibody (Clone MG2B-57, Biolegend) was added to co-cultures, to study NK activating receptor-mediated tumor cell killing mechanism.

#### *In vitro* mixed lymphocyte reaction (MLR) assay: Studying ^3rd^HSC-iNKT cell inhibition of allogeneic T Cell response

PBMCs of multiple healthy donors were irradiated at 2,500 rads and used as stimulators, and non-irradiated allogeneic PBMCs were used as responders. To separate the different donor PBMCs when performing flow cytometry, HLA-A2^+^ responders and HLA-A2^-^ stimulators were used in this study. Irradiated stimulators (2.5 × 10^5^ cells/well) and responders (1 × 10^4^ cells/well) were co-cultured with or without the addition of ^3rd^HSC-iNKT cells (1 × 10^4^ cells/well) in 96-well round bottom plates in C10 medium for up to 4 days. For detection of composition and phenotype using flow cytometry, cells were collected on day 1. For IFN-γ production using ELISA, cell culture supernatants were collected on day 4. To study CD1d-dependent killing mechanism of ^3rd^HSC-iNKT cells, 10 μg/mL of LEAF^TM^ purified anti-human CD1d (Clone 51.1, Biolegend) or LEAF^TM^ purified mouse lgG2bk isotype control antibody (Clone MG2B-57, Biolegend) was added to co-cultures.

#### Bioluminescence live animal imaging (BLI)

BLI was performed using a Spectral Advanced Molecular Imaging (AMI) HTX imaging system (Spectral instrument Imaging). Live animal imaging was acquired 5 min after intraperitoneal (i.p.) injection of D-Luciferin (1 mg per mouse). Imaging results were analyzed using an AURA imaging software (Spectral Instrument Imaging).

#### Human PBMC xenograft NSG mouse model: Studying ^3rd^HSC-iNKT cell amelioration of GvHD

NSG mice were pre-conditioned with 100 rads of total body irradiation (day −1), followed by intravenous injection of 2 × 10^7^healthy donor PBMCs with or without the addition of 2 × 10^7 3rd^HSC-iNKT cells. Mice were weighed daily, bled weekly, and scored 0–2 per clinical sign of GvHD (i.e., body weight, activity, posture, skin thickening, diarrhea, and dishevelment). Mice were terminated and analyzed when moribund. Various mouse tissues (i.e., blood, spleen, liver, lungs, bone marrow, skin, and salivary ligand) were harvested and processed for either flow cytometry or histologic analysis.

#### Human PBMC xenograft NSG mouse model: Studying CD14^+^ myeloid cell modulation of GvHD

NSG mice were pre-conditioned with 100 rads of total body irradiation (day −1), followed by intravenous injection of 2 × 10^7^healthy donor PBMCs or 9 × 10^6^ CD14-depleted donor PBMCs. The amount of PBMCs given was normalized to contain the same number of T cells. Mice were weighed daily, bled weekly, and scored 0–2 per clinical sign of GvHD (i.e., body weight, activity, posture, skin thickening, diarrhea, and dishevelment).

#### Human CD14^−^Depleted PBMC xenograft NSG mouse model: Studying ^3rd^HSC-iNKT cell amelioration of GvHD

NSG mice were pre-conditioned with 100 rads of total body irradiation (day −1), followed by intravenous injection of 9 × 10^6^ CD14-depleted donor PBMCs with or without the addition of 2 × 10^7 3rd^HSC-iNKT cells. Mice were weighed daily, bled weekly, and scored 0–2 per clinical sign of GvHD (i.e., body weight, activity, posture, skin thickening, diarrhea, and dishevelment). Mice were terminated and analyzed when moribund.

#### Raji-FG human B cell lymphoma xenograft NSG mouse model: Studying ^3rd^HSC-iNKT cell retention of GvL effect

NSG mice were pre-conditioned with 100 rads of total body irradiation (day −1), followed by subcutaneous inoculation with 1 × 10^5^ Raji-FG cells (day 0). On day 3, the tumor-bearing experimental mice received intravenous (i.v.) injection of 2 × 10^7^healthy donor PBMCs with or without the addition of 2 × 10^7 3rd^HSC-iNKT cells. Tumor load were monitored over time using BLI. Mice were also weighed daily, bled weekly, and scored 0–2 per clinical sign of GvHD (i.e., body weight, activity, posture, skin thickening, diarrhea, and dishevelment). Mice were terminated and analyzed when moribund.

#### HL60-FG human acute myeloid leukemia xenograft NSG mouse model: Studying ^3rd^HSC-iNKT cell retention of GvL effect

NSG mice were pre-conditioned with 175 rads of total body irradiation (day −1), followed by intravenous inoculation with 2 × 10^5^ HL60-FG (day 0). On day 3, the tumor-bearing experimental mice received intravenous (i.v.) injection of 2 × 10^7^healthy donor PBMCs with or without the addition of 2 × 10^7 3rd^HSC-iNKT cells. Tumor load were monitored over time using BLI. Mice were also weighed daily, bled weekly, and scored 0–2 per clinical sign of GvHD (i.e., body weight, activity, posture, skin thickening, diarrhea, and dishevelment). Mice were terminated and analyzed when moribund.

#### Histological analysis

Tissues (i.e., liver, lungs, salivary glands, and skin) were collected from the experimental mice, fixed in 10% Neutral Buffered Formalin for up to 36 h, then embedded in paraffin for sectioning (5 μm thickness). Tissue sections were prepared and stained with Hematoxylin and Eosin (H&E) or anti-CD3 by the UCLA Translational Pathology Core Laboratory, following the Core’s standard protocols. The H&E-stained sections were imaged on a Zeiss Observer II upright microscope. All images were captured at either 100 × or 200 × and processed using Zen Blue software. GvHD pathological score was calculated as follows: skin: epidermal changes (0–3), dermal changes (0–3), adipose changes (0–3); salivary: infiltration (0–4), follicular destruction (0–4); liver: duct infiltration (0–3), number of ducts involved (0–3), liver cell apoptosis (0–3); lung: infiltrates (0–3); pneumonitis (0–3), overall appearance (0–3). For CD3 surface area measurements, the anti-CD3-stained sections were scanned in their entirety using Hamamatsu Nanozoomer 2.0 HT. The % CD3^+^ area was determined by CD3^+^ area divided by total tissue area, using an Image-ProPremier software.

#### Statistical analysis

GraphPad Prism 6 (Graphpad Software) was used for statistical data analysis. Student’s two-tailed *t* test was used for pairwise comparisons. Ordinary 1-way ANOVA followed by Tukey’s multiple comparisons test was used for multiple comparisons. Log rank (Mantel-Cox) test adjusted for multiple comparisons was used for Meier survival curves analysis. Data are presented as the mean ± SEM, unless otherwise indicated. In all figures and figure legends, “N” represents the number of samples or animals utilized in the indicated experiments. A p value of less than 0.05 was considered significant. ns, not significant; ∗p < 0.05; ∗∗p < 0.01; ∗∗∗p < 0.001; ∗∗∗∗p < 0.0001.

## Data Availability

• All data reported in this manuscript are available from the lead contact without restriction. • No custom computer code was reported in this manuscript. • Any additional information required to reanalyze the data reported in this paper is available from the lead contact on request.
