# Supplementary material for: Off-the-shelf third-party HSC-engineered iNKT cells for ameliorating GvHD while preserving GvL effect in the treatment of blood cancers
Source: iScience. 2022 Aug 6;25(9):104859. doi: 10.1016/j.isci.2022.104859 (PMC9399487; doi:10.1016/j.isci.2022.104859)
Supplement: Document S1. Figures S1–S5 [file mmc1.pdf]

iScience, Volume *25*

Supplemental information

Off-the-shelf third-party HSC-engineered iNKT cells for ameliorating GvHD while preserving GvL effect in the treatment of blood cancers

Yan-Ruide Li, Samuel Zeng, Zachary Spencer Dunn, Yang Zhou, Zhe Li, Jiaji Yu, Yu-Chen Wang, Josh Ku, Noah Cook, Adam Kramer, and Lili Yang

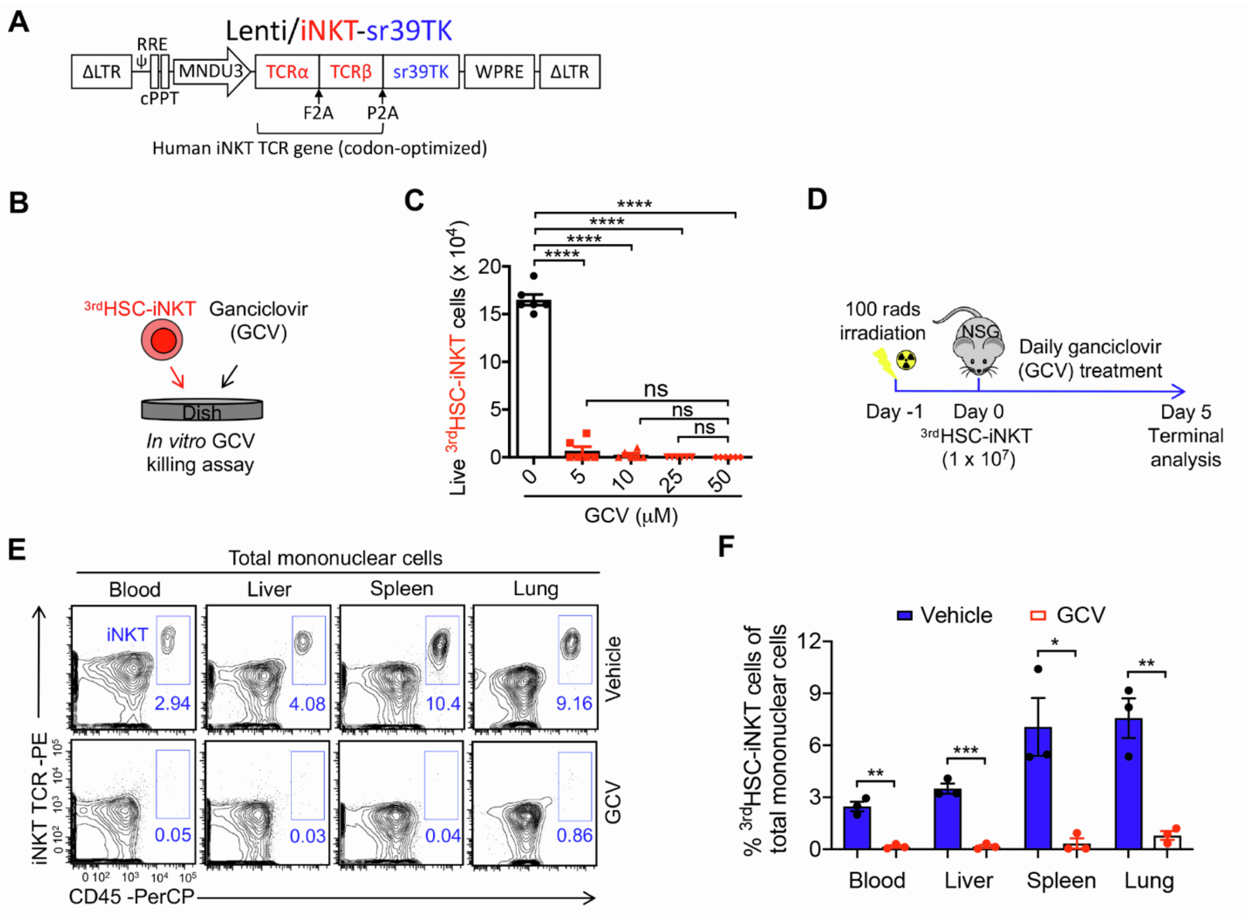

Fig. S1: Controlled depletion of HSC-iNKT cells; related to Fig. 1.

(A) Schematics showing the design of Lenti/iNKT-sr39TK vector. LTR, long terminal repeat; RRE, Rev responsive element; cPPT, central polypurine tract; F2A, 2A self-cleavage sequence derived from the foot-and-mouth disease virus; P2A, 2A self-cleavage sequence derived from the porcine teschovirus-1; WPRE, woodchuck hepatitis virus post-transcriptional regulatory element.

(B-C) *In vitro* controlled depletion of HSC-iNKT cells via GCV treatment. GCV, ganciclovir. (B) Experimental design. (C) Quantification of live cells via cell counting. N = 6. (D-F) *In vivo* controlled depletion of HSC-iNKT cells via GCV treatment. (D) Experimental design. (E) FACS detection of HSC-iNKT cells in the peripheral blood, liver, spleen, and lung of NSG mice at day 5. (F) Quantification of (E). N = 3.

Representative of 2 (D-F) and 3 (B and C) experiments. All data are presented as the mean ± SEM. $^*p < 0.05$, $^{**}p < 0.01$, $^{***}p < 0.001$, and $^{****}p < 0.0001$ by Student's *t* test (F) or by one-way ANOVA (C).

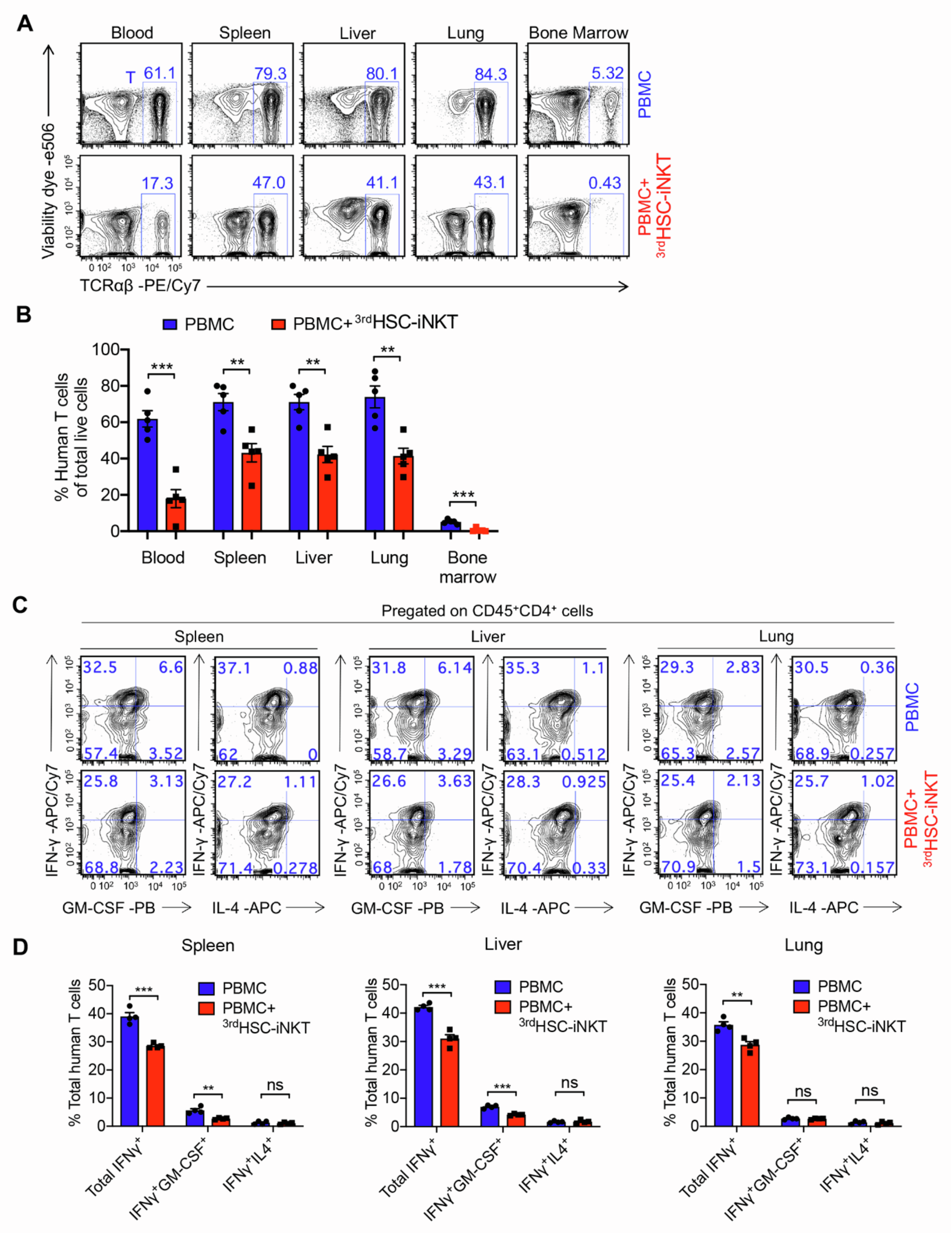

Fig. S2: ^{3rd}HSC-iNKT cells reduce the expansion and Th1 response of donor T cells; related to Fig. 2.

(A) FACS detection of human T cells in the lymphohematopoietic system (i.e., blood, spleen, and bone marrow) and GvHD target organs (i.e., liver and lung) on day 40 after PBMC inoculation. (B) Quantification of (A). N = 5. (C) FACS measurement of cytokine (i.e., IFN-γ, GM-CSF, and IL-4) production of human $CD4^+$ T cells harvested from the spleen, liver, and lung on day 40 after PBMC inoculation. (D) Quantification of (C). N = 4.

Representative of 3 experiments. All data are presented as the mean ± SEM. ns, not significant, $^{**}p < 0.01$ and $^{***}p < 0.001$ by Student's *t* test.

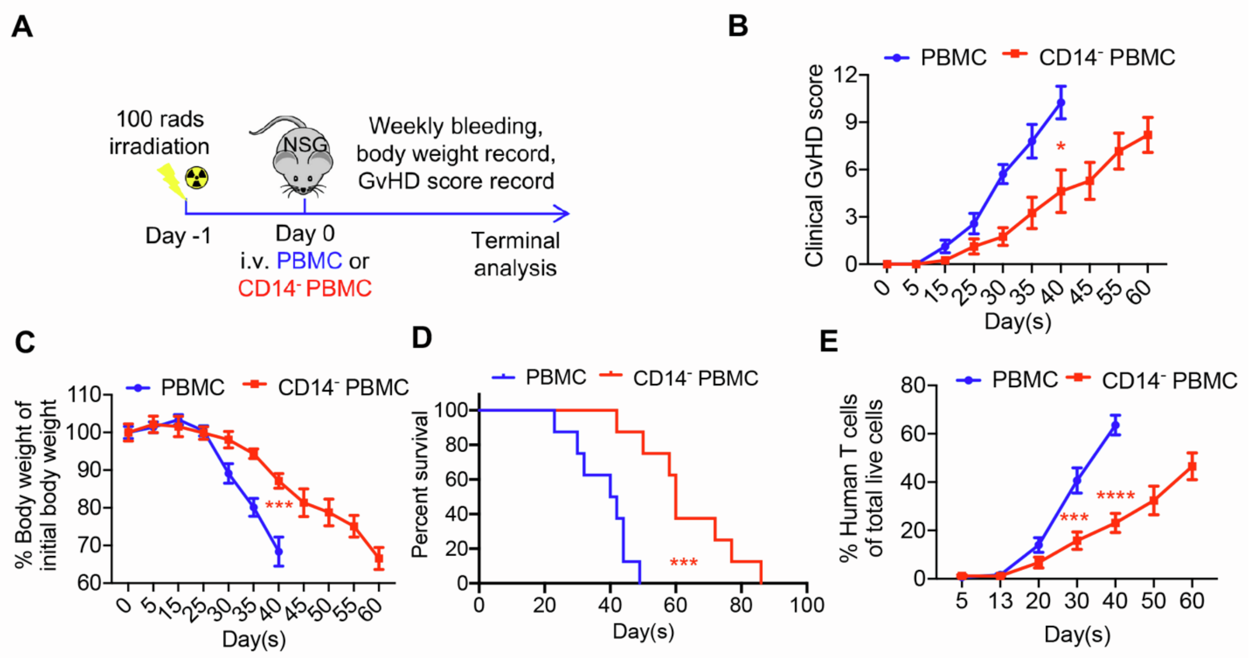

Fig. S3: Donor CD14$^+$ myeloid cells exacerbate GvHD in NSG mice engrafted with human PBMCs; related to Fig. 3.

Sublethally irradiated NSG mice received intravenous injection of 2 x 10^7 healthy donor PBMCs or 9 x 10^6 CD14-depleted PBMCs (normalized to contain the same number of T cells) and were then observed for GvHD development. (A) Experimental design. (B) Clinical GvHD score. (C) Body weight (*p* was calculated using data on day 40). (D) Kaplan-Meier survival curves. (E) Human T cells in peripheral blood of experimental mice over time. N = 8.

Representative of 3 experiments. All data are presented as the mean ± SEM. ns, not significant, *$p < 0.05$, ***$p < 0.001$, and ****$p < 0.0001$, by Student's *t* test (B, C, and E) or by log rank (Mantel-Cox) test adjusted for multiple comparisons (D).

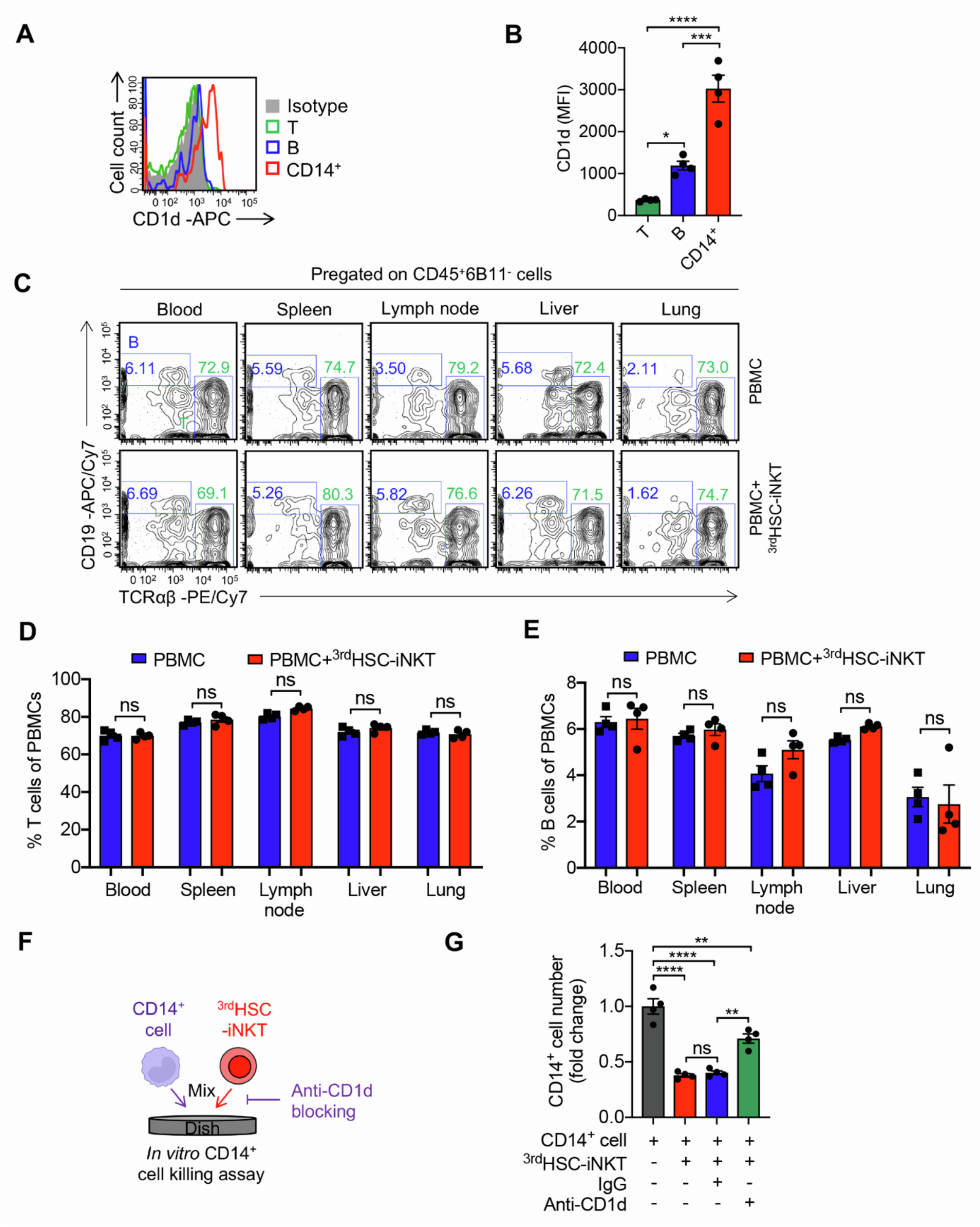

Fig. S4: 3rdHSC-iNKT cells do not deplete donor T and B cells; related to Fig. 3 and 4.

(A-E) Sublethally irradiated NSG mice received intravenous injection of 2 x 10^7 healthy donor PBMCs with or without the addition of 2 x 10^7 ^{3rd}HSC-iNKT cells and were analyzed 3 days later. (A) Comparison of CD1d expression on human $CD14^+$ myeloid, B, and T cells isolated from peripheral blood of recipient animals. (B) Quantification of (A). N = 4. (C) FACS detection of donor T and B cells in the blood, spleen, lymph node, liver and lung of recipient animals. (D and E) Quantification of (C). N = 4.

(F and G) $CD14^+$ myeloid cells were isolated from healthy donor PBMCs and co-cultured with ^{3rd}HSC-iNKT cells for 24 hours ($CD14^+$ myeloid cell:iNKT ratio 1:1). Where applicable, purified anti-human CD1d antibody or its IgG isotype control was also added. (F) Experimental design. (G) Quantification of live $CD14^+$ myeloid cells via flow cytometry. N = 4.

Representative of 3 experiments. All data are presented as the mean ± SEM. ns, not significant, *$p < 0.05$, **$p < 0.01$, ***$p < 0.001$, and ****$p < 0.0001$, by Student's *t* test (D and E) or by one-way ANOVA (B and G).

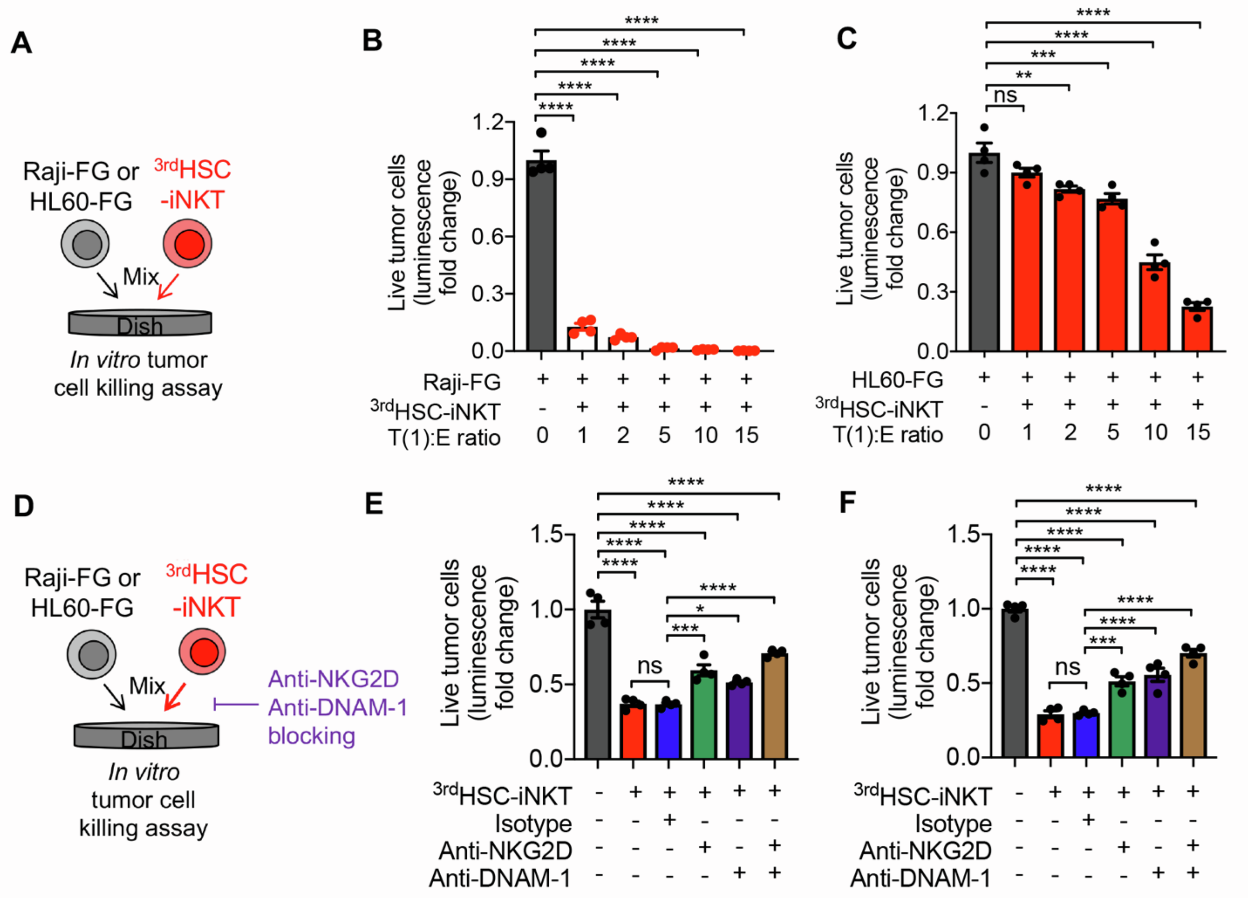

Fig. S5: ^{3rd}HSC-iNKT cells directly target tumor cells through NK function; related to Fig. 5 and 6.

(A-C) Studying the tumor killing efficacy of ^{3rd}HSC-iNKT cells. Two human tumor cell lines were studied: Raji (human B cell lymphoma) and HL60 (human acute myeloid leukemia, AML). (A) Experimental design. (B and C) Tumor killing data of Raji-FG lymphoma and HL60-FG AML cells at 24-hours. N = 4.

(D-F) Studying the tumor killing mechanisms of ^{3rd}HSC-iNKT cells. NKG2D and DNAM-1 mediated pathways were studied. (D) Experimental design. (E) Tumor killing data of Raji-FG lymphoma cells at 24-hours (tumor:iNKT ratio 3:1). N = 4. (F) Tumor killing data of HL60-FG AML cells at 24-hours (tumor:iNKT ratio 1:15). N = 4.

Representative of 3 experiments. All data are presented as the mean ± SEM. ns, not significant, *$p < 0.05$, **$p < 0.01$, ***$p < 0.001$, and ****$p < 0.0001$, by one-way ANOVA.
